# Personality Profiles of Older Suicide Attempters: Cross-Sectional And Prospective Differences From Depressed Nonattempter And Nonpsychiatric Comparisons

**DOI:** 10.1016/j.jagp.2025.05.004

**Published:** 2025-05-07

**Authors:** Anna Szücs, Meghan T. Wong, Emma J. O’Brien, Paulina K. Pankowska, Andrea B. Maier, Katalin Szanto, Hanga Galfalvy

**Affiliations:** Division of Family Medicine, Department of Medicine, Yong Loo Lin School of Medicine, National University of Singapore, Singapore, Singapore; Faculty of Behavioural and Movement Sciences, Vrije Universiteit Amsterdam, Amsterdam, The Netherlands; Department of Psychiatry, School of Medicine, University of Pittsburgh, Pittsburgh, Pennsylvania, USA; Department of Psychiatry, School of Medicine, University of Pittsburgh, Pittsburgh, Pennsylvania, USA; Department of Psychology, Louisiana State University, Baton Rouge, Louisiana, USA; Sociology Department, Utrecht University, Utrecht, The Netherlands; Faculty of Behavioural and Movement Sciences, Vrije Universiteit Amsterdam, Amsterdam, The Netherlands; Academy for Healthy Longevity, Yong Loo Lin School of Medicine, National University of Singapore, Singapore, Singapore; Healthy Longevity Translational Research Program, Yong Loo Lin School of Medicine, National University of Singapore, Singapore, Singapore; Department of Psychiatry, School of Medicine, University of Pittsburgh, Pittsburgh, Pennsylvania, USA; Department of Psychiatry, Columbia University, New York, New York, USA

**Keywords:** Big five, intolerance to ambiguity, narcissism, need for closure, old age, personality, suicidal behavior, suicide

## Abstract

**Objectives:**

Associations between personality and suicide risk in later life have been widely inconsistent, as most traits likely only define subpopulations of older attempters. This study aimed to identify prominent attempter profiles and characterize them clinically.

**Design:**

Exploratory study using latent profile analysis (LPA).

**Setting:**

Longitudinal Research Program for Late-Life Suicide, Pittsburgh, USA.

**Participants:**

The sample comprised 285 participants (mean age = 63.2 years, SD = 7.3), of which 109 were suicide attempters, 111 depressed nonattempters, and 65 nonpsychiatric comparisons. The personality profiles identified in attempters by LPA were compared to nonattempter groups cross-sectionally (N = 285) and at two-year follow-up (N = 171).

**Measurements:**

The LPA employed seven personality traits: Big Five dimensions, grandiose narcissism, and need for closure (intolerance to ambiguity).

**Results:**

The analysis identified three profiles. “Careless labile” attempters (n = 71) scored highest on neuroticism and lower on conscientiousness than other attempters, and had more borderline traits and childhood trauma than comparisons. “Callous narcissistic” attempters (n = 25) scored highest on grandiose narcissism, and lowest on agreeableness; they had the highest planning score at their most serious attempt. “Rigid extraverted” attempters (n = 13) were characterized by higher need for closure and more extraversion than other depressed groups, and were older at their first attempt than other attempters. At two-year follow-up, rigid extraverted attempters showed more improvement of depression severity but steeper cognitive decline than most other depressed groups.

**Conclusions:**

Attempter profiles differed from each other and nonattempters on several key suicidal, clinical, and prospective health-related characteristics. If replicated, these profiles could help with earlier detection of vulnerability and person-centered suicide prevention.

## INTRODUCTION

Mid- and late-life suicide remains an insufficiently prevented, evitable cause of death in the United States. Men aged 55–64 years and women aged 65–74 years had the largest increase in suicide rates between 2001 and 2021 out of all age groups.^1^ Prevention efforts in depressed older adults have primarily focused on proximal risk factors, such as emotion regulation or social connectedness, which is resource-intensive.^2,3^ Meanwhile, the field’s understanding of constitutional characteristics increasing vulnerability to a suicidal crisis in older age remains limited.

Despite borderline personality representing an important risk factor for suicide all ages confounded,^4^ evidence in older adults suggests that an emotionally labile or interpersonally maladaptive personality, as evidenced by borderline traits, high neuroticism, or low extraversion, only characterizes attempters who were already suicidal earlier in life but not those who become suicidal after 50 years of age.^5^ Furthermore, even in younger age groups, associations between the above traits and suicidal behavior remain inconsistent.^6^

Personality traits related to aggressivity, such as antisocial or dissocial traits and high antagonism (measured by low agreeableness), have been linked to an early onset of suicidal behavior and to death by suicide in middle-aged and older individuals.^5,7,8^ Yet, other studies associated avoidant personality and modesty (an agreeableness subdimension) with suicidal behavior or death by suicide,^4,9^ including in older age.^10,11^

Traits indicative of a more conscientious, anankastic, controlling, or rigid personality have been more consistently associated with suicidal behavior or death by suicide occurring in the second half of life in both quantitative^5,10,12^ and qualitative^13,14^ studies. In the case of suicide decedents with such profiles, relatives described a possible intolerance to the prospect of losing control over life due to severe illness (potential cancer) or aging-related decline.^13,15^ In suicide attempters, higher conscientiousness was also associated with higher suicidal intent.^12^ Other studies found middle-aged and older suicide decedents to have lower openness to experience than younger suicide decedents^7,16^ and than similarly aged sudden death and living comparisons,^7,16^ possibly suggesting the presence of other forms of rigidity, namely narrowmindedness and a need for routine, in certain older individuals with lethal attempts.^17^

Finally, in the psychoanalytical literature, multiple authors have linked pathological narcissism, in particular its grandiose dimension, to suicidal behavior related to the aging process and aimed at protecting one’s idealized self-image from decline.^18,19^ Quantitative evidence associating grandiose narcissism with suicidal behavior remains inconclusive.^20^ In actively suicidal younger samples, however, grandiose narcissistic traits have been associated with particularly high-lethality suicidal acts^21^ and pathological narcissism with more suicidal planning^22^ and higher expected lethality of attempts.^23^

Taken together, the above evidence suggests that no single personality profile characterizes all older suicide attempters and decedents. Instead, different subpopulations likely have different profiles and may be characterized by different patterns of suicidal behavior, such as lower versus higher lethality attempts or an earlier versus later onset of suicidal behavior. These subpopulations may additionally be vulnerable to different stressors, as risk factors of late-life suicidal behavior have been found to span a wide range of domains, including mental, cognitive, physical, and social health.^24^ Yet, we lack understanding of which personality traits co-occur and whether they relate to suicidal characteristics and risk factors in older suicide attempters. To our knowledge, no study to date used algorithmic clustering methods to parse personality heterogeneity in suicide attempters. Exploring personality profiles in an older sample allows to contrast subpopulations struggling with suicidal crises from early on with those who became suicidal only later in life.^25^

The present study aimed to identify different subpopulations of older adults with distinct suicidal behavior and individual characteristics using personality traits as a starting point. Employing latent profile analysis (LPA), the study explored:

(Aim 1) Whether combinations of personality traits delineated attempter profiles differing from each other on suicidal behavior characteristics;

(Aim 2) Whether these attempter profiles differed from each other and nonsuicidal depressed and nonpsychiatric comparisons on socio-demographic characteristics as well as on indicators of mental, cognitive, physical, and social health measured cross-sectionally;

(Aim 3) Whether the attempter profiles differed from each other or from comparison groups prospectively in having reattempted suicide, or with respect to changes in their mental, cognitive, and physical health over two years.

## METHODS

### Participants

The sample consisted of 285 adults aged ≥40 years and enrolled in the Longitudinal Research Program for Late-Life Suicide in Pittsburgh (Pennsylvania, USA), who had completed all personality assessments employed in the LPA. Exclusion criteria for the program include psychosis or mania diagnosed at any point in life, neurological disorders, delirium, electroconvulsive therapy within the past six months, and cognitive deficits evidenced by a score of ≤23 points on the Mini-Mental State Examination (MMSE; Folstein et al., 1975) or by a current diagnosis of dementia. These exclusion criteria were motivated by the program’s primary focus, namely cognition and decision-making.

The sample comprised three groups: 109 suicide attempters, who had made at least one lifetime suicide attempt defined as “self-injurious behavior with intent to die and expectation of death” (O’Carroll et al., 1996), 111 depressed nonattempter comparisons with no lifetime history of suicidal behavior, and 65 nonpsychiatric comparisons with no lifetime history of Axis I psychiatric disorders based on the Structured Clinical Interview for DSM-IV Disorders (SCID; First et al., 1995).

Except for nonpsychiatric comparisons, all participants met criteria for a current major depressive episode on the SCID or had a score of ≥14 points on the Hamilton Depression Rating Scale (HDRS), indicating clinical depression (Hamilton, 1960).

### Procedure

All procedures were in accordance with the University of Pittsburgh’s Institutional Review Board. Participants were enrolled in the study from psychiatric inpatient units, psychiatric outpatient groups, clinics, and private practices, as well as the University of Pittsburgh’s research volunteer registry. The baseline evaluation took place over one or several sessions, over video call or in person per the participant’s preference, and comprised all assessments listed below. Participants were then assessed at three months, and then at yearly follow-ups.

### Assessments

#### Personality assessments

Personality measures considered for inclusion into the LPA are described below. They were selected based on their availability in the sample and association with late-life suicidal behavior or its risk factors in the literature (see Introduction). The process leading to the final selection of personality traits for the LPA is detailed under “Statistical analysis”.

NEO Five-Factor Inventory (NEO-FFI).^26^ The NEO-FFI is a 60-item self-report assessing the five-factor (Big Five) personality dimensions: neuroticism (negative affectivity), extraversion (enthusiasm, gregariousness), openness to experience (intellectual curiosity, novelty-seeking), conscientiousness (meticulousness, achievement orientation), and agreeableness (compassion, politeness). Items are assessed on a five-point Likert scale where 0 is “strongly disagree” and 4 is “strongly agree”.

Brief Pathological Narcissism Inventory—Grandiose Narcissism Subscale (BPNI-G).^27^ The BPNI is a 28-item version of the Pathological Narcissism Inventory, a self-report assessing narcissistic grandiosity and vulnerability. Items are scored on a six-point Likert scale ranging from 0 (“Not like me at all”) to 5 (“Very much like me”). Only the grandiosity subscale of the BPNI (BPNI-G) was utilized in the study, to minimize overlap with neuroticism.^27^ Grandiose narcissism is characterized by an inflated ego and sense of one’s capabilities and by interpersonal dominance.

Need for Closure Scale—Brief version (NFC).^28^ The NFC is a 15-item version self-report measuring one’s degree of intolerance to ambiguous or inconclusive outcomes. Each item is rated on a six-point Likert scale, ranging from 1 (“completely disagree”) to 6 (“completely agree”). Higher NFC scores indicate higher levels of trait rigidity with a stronger need for order, predictability, and decisiveness, and greater discomfort with ambiguity.^28^

Personality Assessment Inventory—Borderline Scale (PAI-BOR).^29^ The PAI-BOR is a self-report measuring borderline personality traits, including affective instability, identity problems, negative relationships, and self-harm behaviors. The total score ranges from 0 to 72 points, with higher scores indicating more severe borderline personality. A score of ≥38 points has been used as a clinical cutoff for borderline personality pathology.

#### Suicidal behavior characteristics measured in attempters (Aim 1)

Cross-sectional suicidal behavior characterization included attempters’ total number of lifetime attempts, their age at their first and most recent attempt, and suicidal intent, degree of planning, and medical seriousness (lethality) scores at their most lethal attempt. See Supplementary Table S1 for details.

#### Cross-sectional clinical characterization (Aim 2)

Cross-sectional assessments in all groups encompassed age, race, sex, years of education, per capita income, cognition, executive functioning, impulsivity, physical illness burden, childhood trauma, and social connectedness. Further, depressed groups (attempters and depressed nonattempters) were also assessed for depression severity using the HDRS and for suicidal ideation severity using the Beck Scale for Suicidal Ideation (SSI).^30^ See Supplementary Table S1 for details.

#### Prospective clinical characterization (Aim 3)

Prospective analysis in depressed groups employed five follow-up measures, namely having reattempted during follow-up (for attempters), suicidal ideation severity (SSI), depression severity (HDRS), physical illness burden, as measured by the Cumulative Illness Rating Scale—Geriatrics (CIRS-G),^31^ and cognitive functioning (MMSE). We chose to look at two-year follow-up as it was the latest visit with available data for enough attempter participants (n = 91 with >10 attempters in each profile).

### Statistical Analysis

#### Variable and model selection

Eight personality traits, namely NEO-FFI dimensions, grandiose narcissism (BPNI-G), need for closure (NFC), and borderline traits (PAI-BOR) were considered for inclusion into the LPA. As we aimed to select personality traits that would capture distinct forms of vulnerability and characterize specific subpopulations of attempters, variable selection was based on (a) the traits’ association with specific late-life suicidal behavior characteristics or specific risk factors for late-life suicidal behavior in the literature; (b) a lack of strong intercorrelations between selected traits (Supplementary Figure S1); and (c) indication that the selected traits were more likely to characterize a specific subgroup of attempters than all attempters, i.e., that they would not differentiate attempters as a single group from both nonattempter comparison groups in linear regression models predicting personality traits with study groups, co-varying for age and sex, and employing Bonferroni correction for a set of eight tests to further increase their robustness (Supplementary Table S2). Supplementary Table S3 presents a detailed integration of points (a) to (c).

The LPA, conducted in attempters (n = 109) used the package tidyLPA^32^ in R version 3.6.1. The final model selection with regards to the variance-covariance parameterization and the optimal number of profiles relied on the Bayesian Information Criterion (BIC) and the Akaike Information Criterion (AIC), two goodness-of-fit indicators used to compare nested models, with lower values indicating better fit. We ensured that the selected solution had an entropy ≥0.8, indicating that the obtained profiles were highly discriminative.^33^ We performed a bootstrapped likelihood ratio test to determine whether the number of profiles selected fit the data better than one less number of profiles. We tested the robustness of our model selection process by assessing the number of clusters obtained with k-means clustering applied to the same dataset.

#### Identification of attempter personality profiles and associated suicidal behavior characteristics (Aim 1)

Using one-way ANOVA and Kruskal Wallis test, we investigated differences between the attempter profiles resulting from the LPA and six cross-sectionally measured suicidal behavior characteristics, employing Benjamini-Hochberg adjustment for false discovery rate and Tukey’s Honestly Significant Difference (HSD) for post-hoc pairwise comparisons.

#### Broader cross-sectional characterization of personality profiles (Aim 2)

To further contextualize attempter profile characteristics, we performed 29 descriptive bivariate tests to contrast the profiles with each other and nonattempter comparison groups on variables pertaining to personality, socio-demographics, as well as different aspects of mental, cognitive, physical, and social health, which have been associated with an increased risk of suicidal behavior in later life in the literature (see Supplementary Table S3—last column for expected associations between personality traits entered in the LPA and these variables). We used one-way ANOVA or Kruskal-Wallis test for continuous variables, and chi-square or Fisher’s exact test for categorical variables. Tests were adjusted for false discovery rate with Benjamini-Hochberg. Significant ANOVA and Kruskal-Wallis tests were followed by pairwise comparisons using Tukey’s HSD or Holm’s method, respectively. Under main results, we only report associations that reached a large effect size (Cohen’s f ≥0.40 or *η*^2^ ≥0.14 or Cramer’s V ≥0.60).^34,35^ Supplementary Table S7 presents all results.

#### Prospective change in clinical variables at two-year follow-up (Aim 3)

Suicide risk and other health outcomes were assessed prospectively in the depressed groups, as follow-up data was not routinely collected from nonpsychiatric comparisons. Reattempting was the main prospective outcome (Supplementary Table S3—last column) and was coded as any suicide attempt during the two-year follow-up vs none. Evolution (in particular, decline) of mental, cognitive, and physical health were secondary prospective outcomes, as indicators of worse prognosis (Supplementary Table S3—last column). They were measured by change scores for worst suicidal ideation since last visit (SSI), depression severity (HRSD), physical illness burden (CIRS-G), and cognitive functioning (MMSE), computed by subtracting the baseline value from the two-year visit value for each of these variables. There was no available prospective variable for social health. Attempter profiles and depressed nonattempters were compared on prospective outcomes using Fisher’s exact test and one-way ANOVA with Tukey’s HSD for post-hoc pairwise comparisons.

#### Adjusted analysis for Aims 1 to 3

As personality traits inherently vary with age and sex,^36-38^ we did not include these covariates in our main analyses. Instead, we conducted additional adjusted analyses for all tests performed under Aims 1 to 3 (Supplementary Tables S8 to S10). For variables measuring cognitive health, we adjusted for education in addition of age and sex, based on recommendations from scale developers and other authors.^39,40^ Adjusted analysis employed ANCOVA when the main analysis used ANOVA, parametric ANCOVA on the ranks for dependent variables with skewed close-to-normal distributions, and a suitable type of logistic regression in all other instances (see table captions for details).

## RESULTS

### Sample Characteristics

The sample had a mean age of 63.2 years (SD = 7.3), was 85.6% White and 57.9% female, and had a mean education of 15.2 years (SD = 2.8), corresponding to above high school. The mean HRSD score of depressed nonattempters (17.6 points, SD = 4.6) and attempters (20.3 points, SD = 5.7) indicated moderate levels of depression in both groups. Depressed nonattempters had low to no lifetime suicidal ideation with a median SSI score for the worst lifetime ideation of 2.1 points (SD = 5.2, median = 0, IQR = [0–1]), whereas attempters had a mean score of 11.5 points for their worst ever ideation (SD = 11.1, median = 8, IQR = [1–21]). Attempters’ median number of attempts was one attempt (IQR = 2). On average, attempters were 43 years old (SD = 20.7) at their first attempt and 51 years old (SD = 17.6) at their most recent attempt. Attempers’ age at their first attempt had a bimodal distribution with one peak of first attempts in adolescence and early adulthood, and a second peak of first attempts in late life. Descriptive statistics and histograms of attempters’ age at their first attempt, most recent attempt, and at baseline is presented in Supplementary Table S6.

### Identification of Personality-Based Attempter Profiles

Of the eight personality traits, borderline personality (PAI-BOR scores) was not included in the LPA, given that (a) it has been found to measure general personality pathology rather than a specific personality type,^41^ (b) was strongly correlated with neuroticism (r = 0.72; Supplementary Figure S1), and (c) was the only trait differentiating attempters analyzed as a single group from both depressed nonattempters (B = −5.58, SE = 1.45, t(279) = −3.84, p <0.001) and nonpsychiatric comparisons (B = −23.7, SE = 1.69, t(279) = −14.04, p <0.001) (Supplementary Table S2), further suggesting this trait’s lack of specificity to any distinct attempter subpopulation (Supplementary Table S3).

**FIGURE 1.**
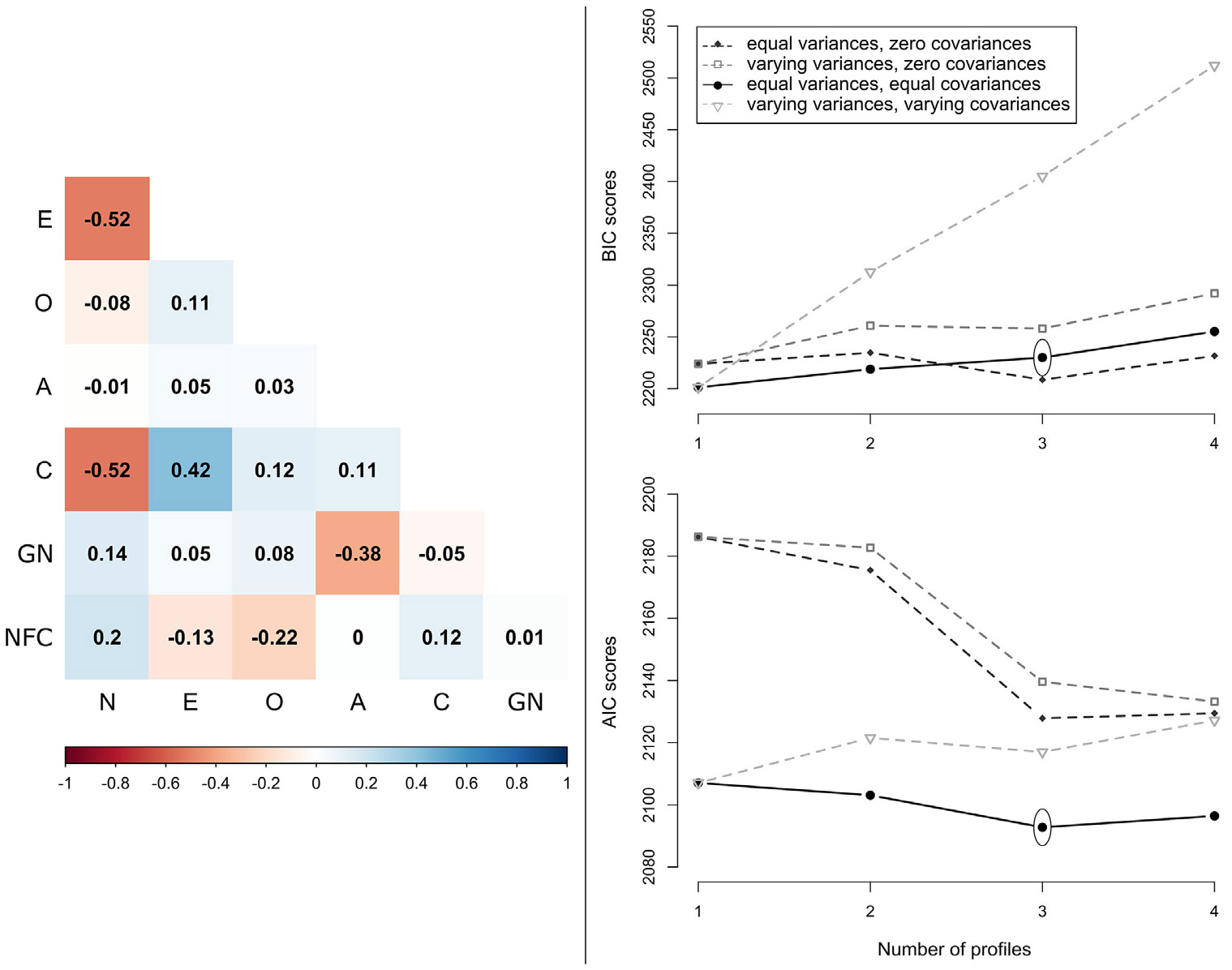
Left panel: Correlations of personality traits included in the latent profile analysis (LPA). Values indicate Pearson correlations. Right panel: LPA estimations for different numbers of profiles and complexity assumptions. Lower scores on both BIC and AIC measures indicate better fit. Ovals indicate the retained solution: three profiles, with equal variances and equal covariances. Legend: N: neuroticism; E: extraversion; O: openness to experience; A: agreeableness; C: conscientiousness; GN: grandiose narcissism; NFC: need for closure; BIC: Bayesian information criterion; AIC: Akaike information criterion.

Correlations between the remaining seven measures, namely the five-factor dimensions (NEO-FFI), grandiose narcissism (BPNI-G), and need for closure (NFC) used in the LPA were small to moderate (Figure 1, left panel). Of all estimated solutions (Figure 1, right panel), the equal variances-equal covariances model with three profiles emerged as the best fitting and most parsimonious across the goodness-of fit-measures considered (Supplementary Table S4). This model also had an acceptable entropy at 0.86, and a posterior probability range between 0.92 and 0.97. It yielded a solution with good clinical face value.

A three-cluster k-means solution for the same set of variables was also recommended based on the average silhouette method and was found acceptable based on the within-cluster sum of squared errors (Supplementary Figure S2). Whereas the three obtained clusters differed from the LPA profiles in size, two clusters had a high stability index (0.86 and 0.84) with their characteristics mapping well on the careless labile and callous narcissistic LPA profiles, as described below (Supplementary Table S5).

### Characterization of Personality Profiles

**FIGURE 2.**
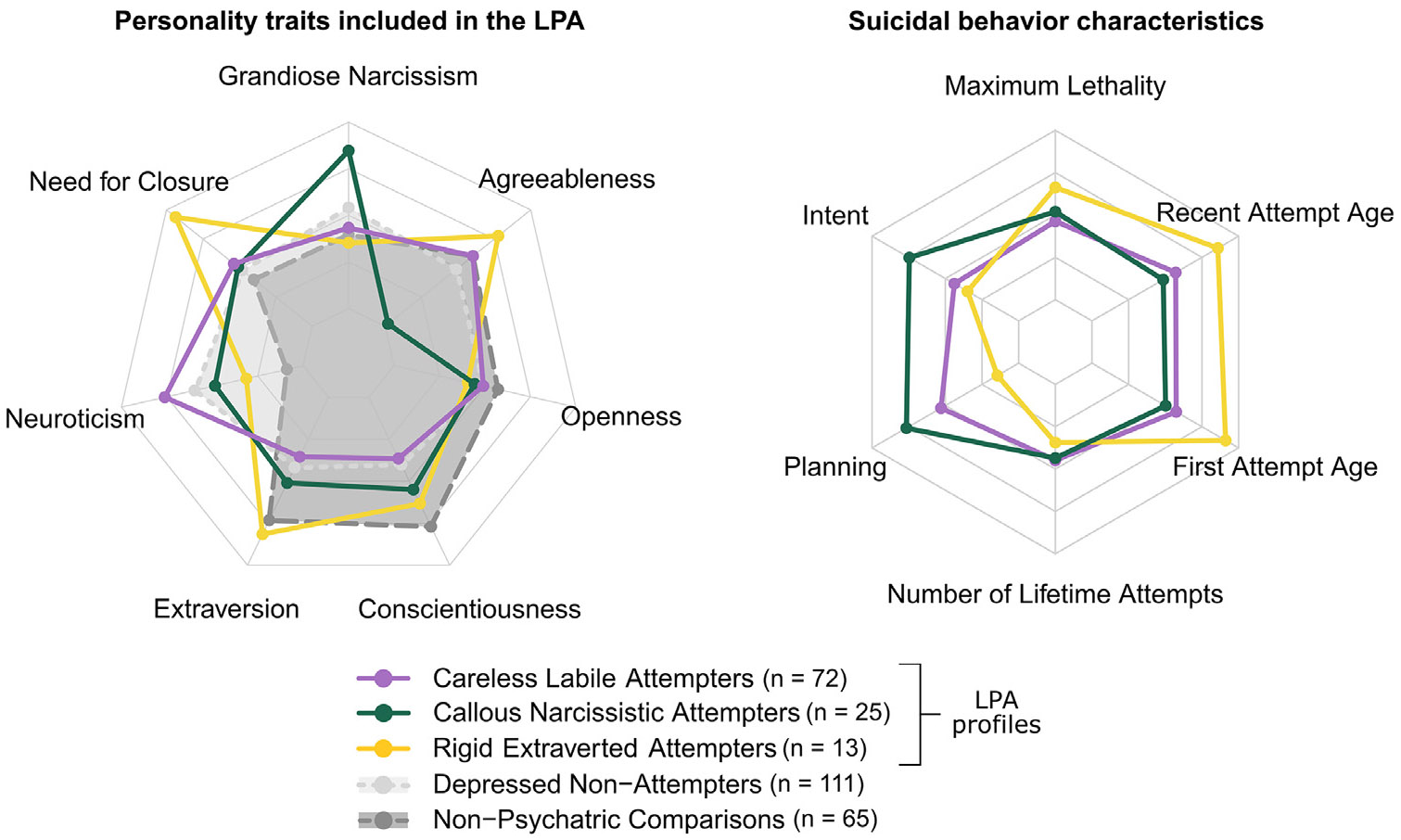
Radar plots of attempter profiles and comparison groups on the personality traits used in the latent profile analysis (LPA) (left) and of attempter profiles on baseline suicidal behavior characteristics (right). The personality profiles were estimated in attempters only (colored lines). On the left radar plot, comparison groups are included for reference (grey surfaces). Points indicate scaled mean scores. LPA: latent profile analysis.

All personality measures entered in the LPA contributed to the differentiation of the profiles, except for openness to experience, which was on average the same across all profiles (Figure 2 and Table 1, upper). Based on the three profiles’ personality characteristics, we labelled them “careless labile” (n = 71), “callous narcissistic” (n = 25), and “rigid extraverted” (n = 13).

The profiles were associated with distinct suicidal behavior characteristics (Table 1, bottom section) and other cross-sectional clinical constructs (Table 2 and Supplementary Table S7) but none was prospectively associated with reattempting during follow-up (Table 3). With respect to basic socio-demographics, the careless labile attempter group had higher proportion of women than the callous narcissistic group, whereas age at baseline did not differentiate the profiles (Table 2).

#### Careless labile attempters

Careless labile attempters (n = 71) scored highest on neuroticism and lowest on conscientiousness of all attempter profiles (Table 1, Figure 1). Their level of suicidal planning was comparable to rigid extraverted attempters’ and they had a similar age of onset of suicidal behavior (40 years) as callous narcissistic attempters (43 years).

In terms of broader clinical characteristics (Table 2), they had higher neuroticism than both comparison groups and lower conscientiousness than nonpsychiatric comparisons. They reported more borderline traits (although not reaching the threshold for borderline personality disorder), higher anger rumination scores, and more childhood trauma than other groups except for callous narcissistic attempters, and higher severity of worst suicidal ideation than depressed nonattempters.

At two years (Table 3), remission from depression and cognitive decline were both less pronounced in careless labile than in rigid extraverted attempters.

#### Callous narcissistic attempters

Callous narcissistic attempters (n = 25) scored highest on grandiose narcissism and lowest on agreeableness of all attempter profiles and reported the highest suicidal planning scores at their most medically serious attempt (Table 1, Figure 1).

With respect to their broader clinical characteristics (Table 2), callous narcissistic attempters scored also higher on grandiose narcissism and lower on agreeableness than comparison groups. They had more borderline traits (although not reaching the threshold for borderline personality disorder) and higher anger rumination scores than rigid extraverted attempters and nonpsychiatric comparisons, more childhood trauma than depressed and nonpsychiatric comparisons, and more severe suicidal ideation than depressed comparisons.

No differences were found between callous narcissistic attempters and other groups at two years follow-up (Table 3).

#### Rigid extraverted attempters

Rigid extraverted attempters (n = 13) scored highest on need for closure and extraversion of all attempter profiles (Table 1, Figure 1). They were the oldest attempter group at their first lifetime attempt (60 years).

Regarding their broader clinical characteristics (Table 2), they were also higher on extraversion than depressed nonattempters and higher on need for closure than both nonsuicidal comparison groups. They had less borderline traits and lower anger rumination scores than other attempters. They had lower cognitive functioning than all other groups except for callous narcissistic attempters.

At two years (Table 3), rigid extraverted attempters experienced a steeper decrease in depression severity than depressed nonattempters and careless labile attempters, although their cognitive functioning also declined more steeply than the latter groups’.

#### Adjusting for socio-demographic covariates

The findings reported above were robust to adjusting for age and sex, as well as education in the case of measures of cognitive health (Supplementary Tables S8 to S10). Due to differences between tests used in the unadjusted and adjusted analyses, the effect size of worst suicidal ideation no longer corresponded to a strong effect size (main analysis: *η*^2^ = 0.27 vs. adjusted analysis: Cramer’s V = 0.43).^34^ However, this difference in effect size did not change with the inclusion or exclusion of covariates.

## DISCUSSION

We identified three personality profiles among older attempters reflecting distinct clinical pictures: careless labile (n = 71), callous narcissistic (n = 25), and rigid extraverted attempters (n = 13). Rigid extraverted attempters had a higher need for closure, but were also more extraverted than other depressed groups. They displayed a steeper cognitive decline at two years than most other depressed groups, yet a more pronounced improvement of their depression severity. Rigid extraverted attempters had the latest onset suicidal behavior (60 years on average). In contrast, careless labile and callous narcissistic attempters had an early onset of suicidal behavior (respectively 40 and 43 years on average), more anger rumination, childhood trauma, and borderline traits than most other groups. From all study groups, careless labile attempters scored highest on neuroticism, whereas callous narcissistic attempters scored highest on grandiose narcissism and lowest on agreeableness. Callous narcissistic attempters reported higher suicidal planning than other attempters.

The above findings confirm that different proximal risk factors predominate in different attempter subpopulations. Dementia prodrome, which has been associated with late-onset suicidal behavior,^42^ may map on the cognitive decline captured in rigid extraverted attempters, who mostly possess adaptive personality traits. These findings also align with the qualitative description of 23 older suicide decedents in Norway, described as controlling and “action-oriented achievers,”^14^ whose suicidal motivation may have arisen from the fear of losing their “freedom of action and self-determination.”^15^ Consistently, the favorable evolution on depression severity of rigid extraverted attempters suggests a suicidal crisis limited in time, consistently with the established peak of suicidal acts during initial stages of dementia.^43^ However, this interpretation needs to be treated with caution given the small size of the rigid extraverted attempter group (n = 13), the presence of baseline cognitive deficits in all depressed groups when considering a cutoff for normal cognitive functioning at 137 points on the DRS,^40^ and the relatively minor change in cognition in rigid extraverted attempters of less than two points on average on the MMSE in two years.

In contrast, careless labile and callous narcissistic attempters may correspond to subpopulations of early-onset attempters who have been encountering acute stressors precipitating suicidal acts since early life or midlife. Prominent risk factors in these groups, such as early adversity, borderline traits, and severe suicidal ideation have been found to facilitate the transition from suicidal thoughts to attempts^44,45^ and have been associated with early-onset suicidal behavior in both younger^46^ and older^5,47^ samples. However, despite sharing several suicide risk factors, careless labile and callous narcissistic attempters had distinct personality profiles, with more internalizing tendencies in careless labile attempters and more externalizing or antagonistic behavioral patterns in callous narcissistic attempters. These results corroborate prior findings reporting that both internalizing and externalizing symptoms increase early suicide risk^48^ and further highlight that both set of symptoms can be carried forward into older age.

The high suicidal planning scores in callous narcissistic attempters corroborate that the classic clinical picture of suicide risk in grandiose narcissism^18^ is likely present in a subpopulation of attempters and may only become apparent to clinicians when evaluating their depressed clients/patients from a personality perspective.

This study is the first with the means to investigate personality profiles bringing together most traits highlighted by the late-life suicide literature in a longitudinal, well-characterized sample of high-suicide-risk older adults. LPA was able to tease apart subpopulations of late-life suicide attempters by identifying profiles that possessed external validity, as they corroborated established suicide risk factors and connected them to consistent clinical pictures. These personality profiles may constitute meaningful distal vulnerability factors for late-life suicide.

Among limitations, we note that small group sizes in the prospective analysis precluded a more conclusive prospective validation of the identified profiles. The differences in size between LPA profiles and k-means clusters further limit their validity and warrants replication in other samples. Some participants may have been subject to recall bias in their documentation of early life events. Due to its recruitment strategy, the study’s sample may have overrepresented attempters with psychiatric diagnoses. As a result, our study may have failed to sample suicidal individuals without a known psychiatric disorder, who may represent a large and possibly distinct subpopulation of attempters.^49^ The present study’s sample was 86% White and 13% Black. Whereas these numbers reflect the race distribution in the Northeastern region of the United States,^50^ they may restrict the generalizability of our findings to other high-income Western regions and countries with similar demographic characteristics. Whereas the study investigated a broad range of suicidal behavior characteristics and suicide risk factors, it may have failed to cover several other clinically relevant ones, for instance the suicidal method used or bereavement preceding the attempt.^24^ Finally, personality assessments were cross-sectional and could not capture possible changes in personality throughout the aging process.^36^

Traditional suicide risk indicators, such suicidal and psychopathological history, have not enabled effective suicide prevention and the field is increasingly turning towards more person-centered, collaborative approaches.^51^ By focusing on general behavioral patterns instead of isolated life events or acute stressors, personality profiling can help clinicians achieve earlier prevention, possibly even before the first occurrence of psychopathology, and better understand patients’ or clients’ needs during counseling or safety planning. The risk profiles found in this study need to be replicated in larger, more socio-demographically diverse samples with a broader range of psychopathology, but nonetheless suggest that personality profiling holds promise.

## Supplementary Material

supplement

Supplementary material associated with this article can be found in the online version at https://doi.org/10.1016/j.jagp.2025.05.004.

## Figures and Tables

**TABLE 1.** Attempter Profile Scores on the Personality Traits Entered in the LPA, Basic Socio-Demographics (Age and Sex), and Suicidal Behavior Characteristics (n = 109)

Attempter Subsample: n = 109	Careless Labile Attempters [CL] n = 71 65.14%	Callous Narcissistic Attempters [CN] n = 25 22.94%	Rigid Extraverted Attempters [RE] n=13 11.92%	Test Statistic F-Statistic Unless Otherwise Specified	Degrees of Freedom	Effect Size Cohen’s f Unless Otherwise Specified	p-Value (Top and Middle Sections) or Adjusted p-Value (Lowest Section)	Posthoc Pairwise Contrasts Adjusted With Tukey Unless Otherwise Specified
Personality Measures (Expressed as Item Means) Used in the LPA								
**Neuroticism**	3.70 (0.79)	2.90 (0.63)	2.40 (0.59)	23.73	2,106	0.67	<0.001	RE,CN<CL
**Extraversion**	2.71 (0.61)	3.02 (0.58)	3.62 (0.67)	12.84	2,106	0.49	<0.001	CL,CN<RE
Openness to experience	3.19 (0.35)	3.14 (0.37)	3.10 (0.33)	0.52	2,106	0.10	0.593	-
**Agreeableness**	3.95 (0.28)	3.40 (0.24)	4.19 (0.20)	51.78	2,106	0.99	<0.001	CN<CL<RE
**Conscientiousness**	3.27 (0.78)	3.68 (0.62)	3.86 (0.54)	5.54	2,106	0.32	0.005	CL<RE,CN
**Grandiose narcissism**	1.61 (0.81)	2.78 (0.80)	1.38 (1.06)	20.19	2,106	0.62	<0.001	CL,RE<CN
**Need for closure**	3.98 (0.73)	3.91 (0.69)	4.88 (0.54)	10.03	2,106	0.44	<0.001	CL,CN<RE
Basic Socio-Demographics								
Age	59.79 (7.78)	61.88 (7.10)	66.15 (9.03)	1.14	2,106	0.15	0.324	-
**Female sex—count (%)**	51 (71.83)	10 (40.00)	7 (53.85)	8.44^a^	2	0.24^c^	0.015	CN<CL^e^
Suicidal Behavior Characteristics								
Maximum lethality of suicidal behavior (BLS); n = 106	3.28 (1.81)	3.50 (2.32)	4.08 (2.40)	0.89	2,103	0.13	1	-
Intent score of the most lethal suicide attempt (SIS); n= 106	17.96 (5.02)	20.96 (4.39)	17.08 (3.75)	4.39	2,103	0.29	0.089	-
**Planning score for the most lethal suicide attempt (SIS Planning subscale); n = 107**	7.64 (2.88)	9.40 (3.10)	5.69 (1.60)	7.81	2,104	0.39	0.004	CL,RE<CN
Number of lifetime attempts—median [IQR]	1 [1–3]	1 [1–3]	1 [1–2]	1.45^b^	2	0.01^d^	0.485	-
**Age at first attempt in years**	40.23 (20.52)	43.24 (18.86)	60.38 (18.01)	5.65	2,106	0.33	0.028	CL,CN<RE
Age at most recent attempt in years	49.15 (17.79)	52.16 (16.71)	64.23 (12.66)	4.32	2,106	0.29	0.095	-

*Note.* Bolded variables significantly differ between groups. Nonbolded variables are nonsignificant and presented only for sample characterization. Columns in grey represent mean (SD) for the three attempter profiles unless otherwise specified. The number of observations is specified under variables with missing data. The p-values of group comparisons on suicidal behavior characteristics (lowest section of the table) have been adjusted for 6 tests using Benjamini-Hochberg’s method. BLS: Beck Lethality scale; LPA: latent profile analysis; SIS: Beck Suicidal Intent Scale.

a Chi-square test value.

b Kruskal Wallis H-statistic.

c Effect size computed as Cramer’s V.

d Effect size computed as *η*^2^.

e Post hoc pairwise contrasts obtained from pairwise chi-square tests adjusted with Bonferroni.

**TABLE 2.** Comparisons Across Attempter Profiles and Nonattempter Groups

Total Sample: N = 285	Careless Labile Attempters [CL] n = 71	Callous Narcissistic Attempters [CN] n = 25	Rigid Extraverted Attempters [RE] n = 13	Depressed NonAttempter Comparisons [DNA] n = 111	Non Psychiatric Comparisons [NPC] n = 65	Test Statistic F-Statistic Unless Otherwise specified	Degrees of Freedom	Effect size Cohen’s f Unless Otherwise specified	Adjusted p-Value	Posthoc Pairwise Contrasts Adjusted With Tukey Unless Otherwise Specified
Personality Measures Used in the LPA										
**Neuroticism (NEO-FFI)**	3.70 (0.79)	2.90 (0.63)	2.40 (0.59)	3.23 (0.68)	1.77 (0.43)	85.22	4,280	1.10	<0.001	NPC<other groups<CL RE<DNA
**Extraversion (NEO-FFI)**	2.71 (0.61)	3.02 (0.58)	3.62 (0.67)	2.84 (0.60)	3.46 (0.50)	20.08	4,280	0.54	<0.001	CL,CN,DNA<RE,NPC
Openness to experience (NEO-FFI)	3.19 (0.35)	3.14 (0.37)	3.10 (0.33)	3.19 (0.37)	3.29 (0.42)	1.26	4, 280	0.13	1	-
**Agreeableness (NEO-FFI)**	3.95 (0.28)	3.40 (0.24)	4.19 (0.20)	3.89 (0.34)	4.01 (0.28)	22.89	4,280	0.57	<0.001	CN<other groups DNA<RE
**Conscientiousness (NEO-FFI)**	3.27 (0.78)	3.68 (0.62)	3.86 (0.54)	3.35 (0.68)	4.16 (0.43)	21.42	4,280	0.55	<0.001	CL<RE,NPC CN,DNA<NPC
**Grandiose narcissism (BPNI-G)**	1.61 (0.81)	2.78 (0.80)	1.38 (1.06)	1.92 (0.93)	1.51 (0.85)	11.62	4,280	0.41	<0.001	other groups<CN NPC<DNA
Need for closure (NFC)	3.98 (0.73)	3.91 (0.69)	4.88 (0.54)	3.85 (0.75)	3.67 (0.75)	8.09	4,280	0.34	<0.001	other groups<RE
Other Personality Measure										
**Borderline traits (PAI-BOR); n = 284**	34.85 (12.69)	31.80 (15.80)	20.00 (11.55)	26.68 (9.42)	8.40 (4.73)	60.92	4,279	0.93	<0.001	RE, DNA<CL RE<CN NPC<other groups
Socio-Demographics										
Age	59.79 (7.78)	61.88 (7.10)	66.15 (9.03)	62.93 (6.79)	63.75 (7.28)	0.80	4, 280	0.11	1	-
Female sex—count (%)	51 (71.83)	10 (40.00)	7 (53.85)	101 (56.11)	76 (54.29)	9.66^a^	4	0.14^c^	1	-
**Per capita yearly Income; n = 274**	19,927 (12,463)	17,859 (11,347)	19,965 (11,870)	25,227 (19,713)	39,527 (17,548)	14.22	4,269	0.46	<0.001	other groups<NPC
Mental Health										
Depression (HDRS); n = 220	20.48 (5.46)	19.24 (5.87)	21.69 (6.68)	17.62 (4.60)	-	5.76	3,216	0.28	0.023	DNA<CL,RE
**Worst suicidal ideation (SSI)—median [IQR]; n = 220**	7 [1–21]	11 [1–21]	0 [0–21]	0 [0–1]	-	60.48^b^	3	0.28^d^	<0.001	DNA<CL,CN^e^
Cognitive Health										
Cognitive functioning – brief assessment (MMSE); n = 278	28.37 (1.53)	27.64 (2.42)	27.85 (1.63)	29.01 (1.06)	29.19 (1.03)	9.18	4,273	0.37	<0.001	attempter profiles<DNA, NPC
**Cognitive functioning—detailed assessment (DRS); n = 262**	136.11 (4.01)	133.50 (4.83)	132.08 (6.37)	136.78 (4.13)	138.97 (2.71)	12.53	4, 257	0.44	<0.001	other groups < NPC RE, CN < DNA RE < CL
**Impulsivity (BIS); n = 281**	70.06 (11.43)	70.08 (12.76)	67.54 (12.68)	65.65 (9.48)	51.89 (7.64)	33.02	4,276	0.69	<0.001	DNA<CL NPC<other groups
**Anger rumination (ARS); n = 284**	38.33 (10.86)	39.20 (11.54)	29.62 (7.03)	35.60 (9.14)	24.48 (4.70)	26.00	4,279	0.61	<0.001	RE,NPC< CL,CN NPC<DNA
Physical Health										
**Physical illness burden (CIRS-G)—median [IQR]; n = 270**	9 [5–13]	8 [5.25–11.75]	9 [6–14.5]	9 [6–13]	4.5 [2–6]	46.88^b^	4	0.17^d^	<0.001	NPC<other groups^e^
Social Health										
**Social network (SNI—Network diversity); median [IQR]; n = 284**	3 [2–5]	4 [2–5]	5 [4–5]	4 [3–6]	6 [5–7]	45.31^b^	4	0.15^d^	<0.001	CL,CN,DNA<NPC^e^
**Childhood Trauma (CTQ)—median [IQR]; n = 269**	51 [37–63.5]	48 [35.5–65]	33.5 [26.75–49.25]	44.5 [33.75–52.25]	28 [25–33]	74.86^b^	4	0.27^d^	<0.001	RE,DNA,NPC<CL DNA<NPC<CN^e^

*Note.* Bolded variables significantly differ between groups. Dark grey columns indicate attempter profiles identified by LPA; light grey columns indicate nonattempter comparison groups. Values in all grey columns represent mean (standard deviation) unless otherwise specified. All p-values have been adjusted for 29 tests using Benjamini-Hochberg’s method. The number of observations is specified under variables with missing data. This table presents variables with group differences having a large effect size (Cohen’s f ≥0.40 or *η*^2^ ≥0.14 or Cramer’s V ≥0.6; bolded), and additional variables important for sample characterization (nonbolded). Other bivariate comparisons are presented in Supplementary Table S7. IQR: interquartile range; NEO-FFI: NEO Five-Factor Inventory; BPNI-G: brief pathological narcissism inventory—grandiosity subscale; PAI-BOR: personality assessment inventory-borderline scale; HDRS: Hamilton rating scale for depression; SSI: beck scale of suicidal ideation; MMSE: mini mental state exam; DRS: global cognitive mattis dementia rating scale; BIS: barratt impulsivity scale; CIRS-G: cumulative illness rating scale—geriatrics; SNI: social network index; CTQ: childhood trauma questionnaire.

a Chi-square test value.

b Kruskal Wallis H-statistic.

c Effect size computed as Cramer’s V.

d Effect size computed as *η*^2^.

e Post hoc pairwise contrasts adjusted with Holm’s method.

**TABLE 3.** Change From Baseline to Two-Years Follow-Up in Suicidal Behavior, Suicidal Ideation Severity, Depression Severity, Physical Illness Burden, and Cognitive Functioning by Depressed Study Groups

Total Sample at Two-Year visit: N = 172	Careless Labile Attempters [CL] n = 61	Callous Narcissistic Attempters [CN] n = 19	Rigid Extraverted Attempters [RE] n = 11	Depressed Nonattempter Comparisons [DNA] n = 81	Test Statistic F-Statistic Unless Otherwise Specified	Degrees of Freedom	Effect Size Cohen’s f Unless Otherwise Specified	p-Value	Posthoc Pairwise Contrasts Adjusted With Tukey
Any follow-up suicidal behavior (vs none)—count (%); n = 91	12 (19.67%)	3 (15.79%)	1 (9.09%)	-	^a^	-	-	0.841	-
Mean change in worst suicidal ideation severity (SSI); n = 140	−3.20 (11.08)	−5.62 (14.79)	−8.83 (11.37)	0.31 (3.87)	4.05	3, 136	0.30	0.009	No pairwise differences
**Mean change in depression severity (HDRS); n = 147**	−6.42 (7.36)	−7.20 (8.35)	−15.40 (9.56)	−5.98 (6.77)	2.71	3, 143	0.24	0.047	RE < CL, DNA
**Mean change in cognitive functioning (MMSE); n = 126**	0.33 (1.58)	−0.27 (2.05)	−1.88 (3.76)	0.28 (0.95)	5.05	3, 122	0.35	0.002	RE < CL, DNA
Mean change in physical illness burden (CIRS-G); n = 132	0.60 (2.73)	1.46 (1.45)	-0.50 (1.64)	1.08 (2.73)	1.08	3, 128	0.16	0.362	-

*Note.* Bolded variables significantly differ between groups. Dark grey columns indicate attempter profiles identified by LPA within the attempter group; the light grey column indicates the depressed nonattempter comparison group. Numbers in the grey cells represent mean (SD) unless otherwise specified. Variables with significant pairwise group differences are bolded. Positive scores indicate an increase from baseline and negative scores a decrease from baseline. Higher scores indicate more unfavorable outcomes for all variables except for cognitive functioning. Number of observations is indicated for each variable as follow-up assessments were missing in some participants. SSI: beck scale of suicidal ideation; HDRS: Hamilton rating scale for depression; CIRS-G: cumulative illness rating scale—geriatrics; MMSE: mini mental state exam.

a Fisher’s exact test (this test has no test statistic).

## Data Availability

Data is available from the authors upon reasonable request.

## Highlights

### • What is the primary question addressed by this study?

This study explores whether specific personality profiles characterize different subpopulations of older suicide attempters.

### • What is the main finding of this study?

Three attempter profiles were identified by latent profile analysis and labelled: *careless labile, callous narcissistic*, and *rigid extraverted*. In bivariate analysis, these profiles differed from each other and from nonattempter comparison groups in cross-sectional and prospective clinical characteristics.

### • What is the meaning of the finding?

Understanding and assessing personality profiles instead of individual traits can help differentiate attempter subpopulations, thus shifting clinical management of suicide risk towards earlier prevention and more person-centered treatment.
