# Supplementary material for: Personality Profiles of Older Suicide Attempters: Cross-Sectional And Prospective Differences From Depressed Nonattempter And Nonpsychiatric Comparisons
Source: Am J Geriatr Psychiatry. Author manuscript; Available in PMC 2025 Jul 22. (PMC12281016; doi:10.1016/j.jagp.2025.05.004)
Supplement: supplement [file NIHMS2095605-supplement-supplement.pdf]

# Supplemental Material

## Supplemental Table S1

*Description of assessments used for group comparisons*

| Construct and scale name and reference | Description of measured construct |
|---|---|
| **Socio-demographics** | |
| Age, sex, race, years of education, marital status, per capita yearly income | |
| **Suicidal behavior characteristics (in attempter profiles)** | |
| **Lethality of the most severe suicide attempt**<br>Beck Lethality Scale (BLS; Beck et al., 1975) | The BLS is a clinician-administered scale used in this study to assess the medical seriousness (lethality) of the most severe suicide attempt, which ranged from 0 (none or little) to 8 (attempt resulting in death). Attempts that are severe enough to require hospitalization are scored ≥4 and are considered high lethality. |
| **Suicide intent and suicidal planning of most severe suicide attempt**<br>Scale of Suicidal Intent (SIS; Beck et al., 1974) | The SIS is a clinician-administered scale assessing the degree of planning of the attempt and the participant's intent to make a suicide attempt. The scale comprises 15 items with all items scored on a scale from 0 to 2 (2 representing maximum severity). For the present study, we report the planning subscale and total score of the SIS for the most severe suicide attempt. |
| **Age at first attempt** | The age at which individuals engaged in their first experience of suicidal behavior with the intent to die. |
| **Age at most recent attempt** | The age at which individuals engaged in their most recent experience of suicidal behavior with the intent to die. |
| **Number of attempts** | The total number of suicide attempts that an individual has made in their lifetime. |
| **Mental health (in depressed groups)** | |
| **Depression severity**<br>Hamilton Rating Scale for Depression (HDRS; Hamilton, 1960) | The HDRS is a clinician-administered 17-item assessment of the severity of current depressive symptoms. The suicidal ideation item (Item 3) measuring suicidal ideation was excluded from total scores to prevent overlaps with the ideation assessment below. Higher scores on the HRSD indicate more severe depression. |
| **Suicidal ideation severity**<br>Beck Scale for Suicidal Ideation<br>(SSI; Beck et al., 1979) | The SSI is a clinician-administered 19-item scale employed to measure the severity of recent (past week or past month) and worst lifetime suicidal ideation. The SSI questionnaire is clinician-administered and includes The 19 items assess levels and severity of suicidal contemplation and planning of a suicide attempt and are rated on a three-point scale from 0 (item not present) to 2 (item strongly present). Total scores range from 0 to 38 with higher scores indicating more severe ideation. |
| **Age of onset of the first depressive episode**<br><br>**Lifetime and baseline substance use disorder**<br>Structure Clinical Interview for DSM-IV Disorder (SCID; First et al., 1995) | The SCID was used to characterize age of onset of the first depressive episode and substance use disorder. The SCID is a clinician-administered diagnostic assessment using the diagnostic criteria included in the American Psychiatric Association's Diagnostic and Statistical Manual for Mental Disorders (DSM) for mental disorders. For lifetime and baseline substance use disorder, the following substances were investigated: alcohol, sedatives/hypnotics/anxiolytics, cannabis, stimulants, opioids, cocaine, hallucinogens/PCP, and "other substances" (i.e., inhalants, non-prescribed sleep or diet medication). |

| Cognitive health | |
|---|---|
| **Global cognitive functioning**<br>Mini Mental State Examination (MMSE; Folstein et al., 1975) | The MMSE is a clinician administered, 11-item assessment tool systematically and thoroughly measuring mental status in five areas of cognitive function: orientation, registration, attention and calculation, recall, and language. Scores range from 0-30, where higher scores indicate higher cognitive function. In clinical settings, a score of 23 or lower is indicative of cognitive impairment. |
| **Global cognitive functioning**<br>Mattis Dementia Rating Scale<br>(DRS; Mattis, 1988) | The DRS assesses global cognitive ability, through five cognitive domains, namely Initiation/Perseveration, Attention, Construction, Conceptualization, and Memory. Only total scores were used in the current analysis and range from 0 to 144, with higher scores indicating better cognitive performance. A cutoff of 137 has been used in clinical settings as a threshold for normal cognitive functioning (Jurica et al., 2001). |
| **Executive functioning**<br>The Executive Interview<br>(EXIT; Royall et al., 1992) | The EXIT is a clinician-administered, 25-item assessment tool having the objectives of evaluating the degree of executive impairment among older adults. Higher scores indicate greater executive impairment. Each item is scored from a minimum of 0 ("intact control") to 2 points ("specific incorrect response or inability to complete the task"), with a total score range of 0 to 50, with higher scores indicating more executive dysfunction. |
| **Impulsivity**<br>Barratt Impulsivity Scale<br>(BIS; Patton et al., 1995) | The BIS is a self-report measure that assesses impulsivity using 30 items that measure different aspects of impulsivity, such as attentional, motor, and non-planning impulsiveness. Participants rate each item on a 4-point scale based on how well it describes them (rarely/never, occasionally, often, almost always/always). Higher BIS total scores indicate higher levels of impulsivity. |
| **Anger rumination**<br>Anger Rumination Scale<br>(ARS; Sukhodolsky et al., 2001) | The ARS is a self-report questionnaire that measures the tendency of individuals to engage in rumination, specifically related to anger. The ARS consists of 19 items that assess various aspects of anger rumination, such as the frequency and duration of angry thoughts, the extent to which these thoughts are perceived as helpful or harmful, and the individual's ability to control or stop these thoughts. Higher scores indicate a stronger tendency to engage in anger rumination. |
| **Physical health** | |
| **Physical illness burden**<br>Cumulative Illness Rating Scale - Geriatric (CIRS-G; Miller et al., 1992) | The CIRS-G is a clinician-administered assessment that measures the medical comorbidity burden in geriatric populations. It evaluates 14 organ systems for the presence and severity of chronic medical conditions. Each system is rated from 0 (no problem) to 4 (severe, life-threatening problem), and the scores are summed to provide a score for the patient's comorbidity burden. In this study, total CIRS-G scores were used (ranging from 0 to 52, as the mental health system is not counted) as well as a binary variable of the presence/absence of severe medical conditions (rated 3 or 4 in any system). |
| **Social health** | |
| **Social connectedness**<br>Social Network Index (SNI; Cohen, 1985) | The SNI is an objective measure of three components of social support: 1) Network Diversity (number of active social roles (e.g., as a parent, child, friend) an individual has in relation to someone else with whom they are regularly in contact), 2) People in Network (total number of people in one's network with whom they have regular contact), and 3) Embedded Networks (number of different social groups/facets one is active in (e.g., family, friend groups, religious organizations). |
| **History of childhood abuse or neglect**<br>Childhood Trauma | The CTQ is a retrospective self-report assessment that measures the occurrence and severity of childhood trauma in adults. The CTQ consists of 28 items that ask about different types of childhood trauma, including emotional, physical, and sexual abuse, as |

| | |
|---|---|
| Questionnaire (CTQ; Bernstein et al., 1997) | well as emotional and physical neglect. Participants rate each item on a 5-point scale indicating how often each experience occurred (never true to very often true) and how severe the impact was (none to very severe). The CTQ provides scores for five subscales (emotional abuse, physical abuse, sexual abuse, emotional neglect, and physical neglect) as well as a total score. Only total CTQ scores were used in the current analysis which can range from 25-125 where higher scores indicate more severe trauma exposure. |

**References to Supplemental Table S1**


Beck, A. T., Beck, R., & Kovacs, M. (1975). Classification of suicidal behaviors: I. Quantifying intent and medical lethality. *American Journal of Psychiatry*, *132*(3), Article 3. https://doi.org/10.1176/ajp.132.3.285

Beck, A. T., Kovacs, M., & Weissman, A. (1979). Assessment of suicidal intention: The Scale for Suicide Ideation. *Journal of Consulting and Clinical Psychology*, *47*(2), 343.

Beck, A. T., Schuyler, D., & Herman, I. (1974). Development of suicidal intent scales. In A. T. Beck, H. L. Resnik, & D. J. Lettieri (Eds.), *The prediction of suicide* (pp. 45–56). Charles Press Publishers.

Bernstein, D. P., Ahluvalia, T., Pogge, D., & Handelsman, L. (1997). Validity of the Childhood Trauma Questionnaire in an Adolescent Psychiatric Population. *Journal of the American Academy of Child & Adolescent Psychiatry*, *36*(3), 340–348. https://doi.org/10.1097/00004583-199703000-00012

Cohen, S., R. Mermelstein, et al. (1985). Measuring the functional components of social support. Social support: Theory, research and applications I. G. Sarason and B. Sarason. *The Hague, Martinus Nijhoff*, 73–94.

First, M. Sr., Gibbon, M., & Williams, J. B. W. (1995). *Structured clinical interview for DSM-IV Axis I Disorders—Patient Edition (SCID-I/P). Version 2.0*.

Folstein, M. F., Folstein, S. E., & McHugh, P. R. (1975). “Mini-mental state”. A practical method for grading the cognitive state of patients for the clinician. *Journal of Psychiatric Research*, *12*(3), Article 3. https://doi.org/10.1016/0022-3956(75)90026-6

Hamilton, M. (1960). A rating scale for depression. *Journal of Neurology, Neurosurgery, and Psychiatry*, *23*(1), 56–62. https://doi.org/10.1136/jnnp.23.1.56

Jurica, P. J., Leitten, C. L., & Mattis, S. (2001). *Dementia rating Scale-2: DRS-2: professional manual*. Psychological Assessment Resources.

Mattis, S. (1988). *Demential Rating Scale (DRS): Professional Manual*. Psychological Assessment Resources.

Miller, M. D., Paradis, C. F., Houck, P. R., Mazumdar, S., Stack, J. A., Rifai, A. H., Mulsant, B. H., & Reynolds, 3rd C. F. (1992). Rating chronic medical illness burden in geropsychiatric practice and research: Application of the Cumulative Illness Rating Scale. *Psychiatry Research*, *41*, 237–248.

Patton, J. H., Stanford, M. S., & Barratt, E. S. (1995). Factor structure of the Barratt impulsiveness scale. *Journal of Clinical Psychology*, *51*(6), Article 6.

Royall, D. R., Mahurin, R. K., & Gray, K. (1992). Bedside assessment of executive cognitive impairment: The Executive Interview (EXIT). *Journal of American Geriatric Society*, *40*, 1221–1226.

Sukhodolsky, G. D., Golub, A., & Cromwell, E. N. (2001). Development and validation of the anger rumination scale. *Personality and Individual Differences*, *31*(5), Article 5. https://doi.org/10.1016/S0191-8869(00)00171-9

**Supplemental Figure S1**

*Pearson correlations between all traits considered for inclusion into the LPA*

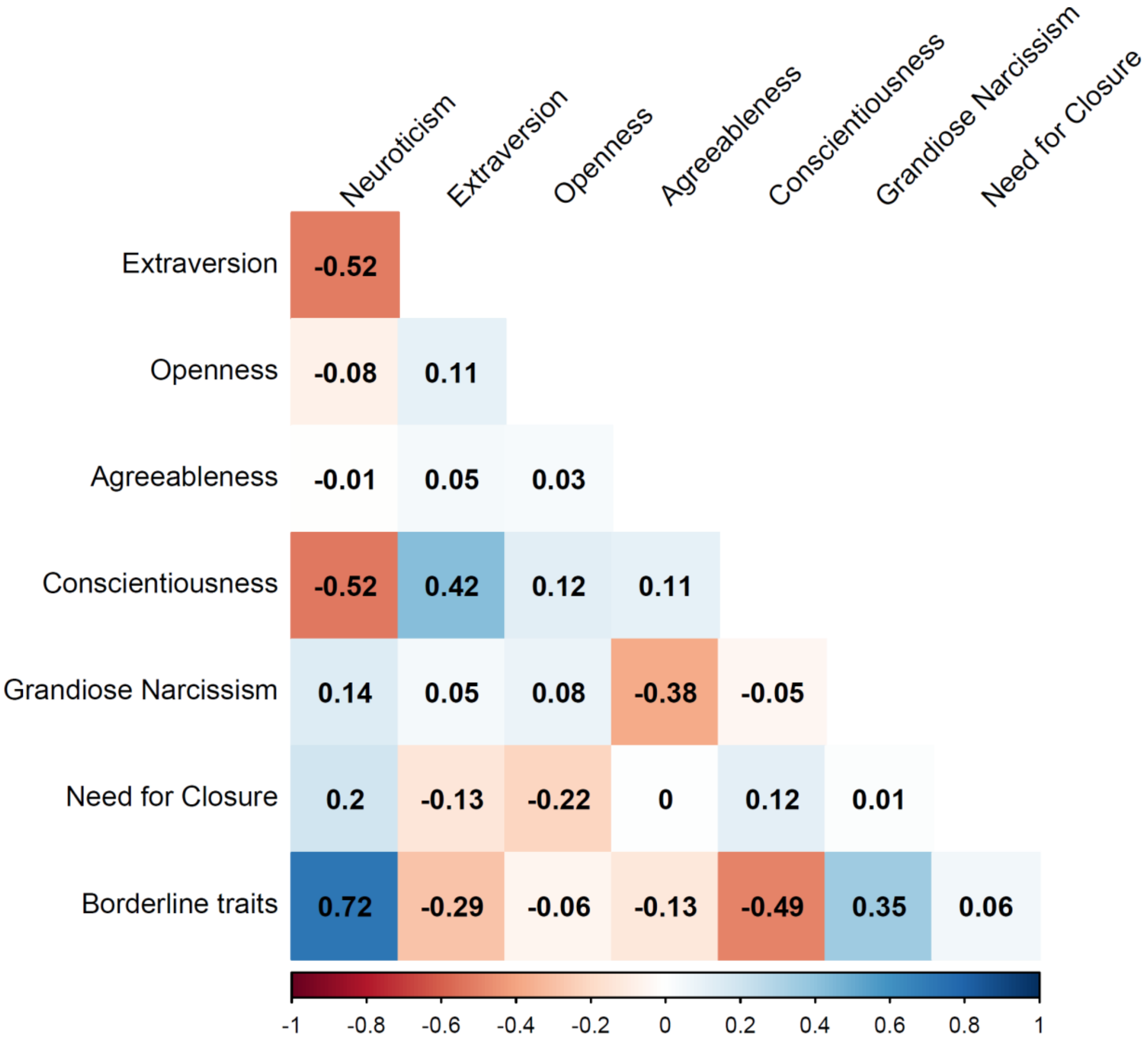

**Supplemental Table S2**

*Covariate-adjusted linear regression models testing differences between attempters and comparison groups on personality traits*

| **Independent variables** | **Regression coefficient** Unstandardized B (standard error) | **t-statistic** | **Residual degrees of freedom** | ***p*-value** | **Adjusted *p*-value** | **Model statistics** |
|---|---|---|---|---|---|---|
| **Linear model predicting Neuroticism** | | | | | | |
| Depressed non-attempters (vs. Attempters) | -0.13 (0.10) | -1.32 | 280 | .187 | 1 | $R^2$ = 0.46<br>Adj. $R^2$ = 0.45<br>F(4,280) = 58.98<br>*p*-value <.001 |
| Non-psychiatric comparisons (vs. Attempters) | -1.59 (0.11) | -14.17 | | <.001 | <.001 | |
| Age | -0.02 (0.04) | -0.44 | | .663 | 1 | |
| Male sex (vs. Female) | -0.12 (0.09) | -1.43 | | .154 | 1 | |
| **Linear model predicting Extraversion** | | | | | | |
| Depressed non-attempter comparisons (vs. Attempters) | -0.05 (0.08) | -0.61 | 280 | .540 | 1 | $R^2$ = 0.15<br>Adj. $R^2$ = 0.14<br>F(4,280) = 12.11<br>*p*-value <.001 |
| Non-psychiatric comparisons (vs. Attempters) | 0.56 (0.10) | 5.87 | | <.001 | <.001 | |
| Age | 0.03 (0.04) | 0.95 | | .345 | 1 | |
| Male sex (vs. female) | -0.02 (0.07) | -0.28 | | .777 | 1 | |
| **Linear model predicting Openness to experience** | | | | | | |
| Depressed non-attempter comparisons (vs. Attempters) | 0.02 (0.05) | 0.36 | 280 | .722 | 1 | $R^2$ = 0.02<br>Adj. $R^2$ = 0.01<br>F(4,280) = 1.47<br>*p*-value = .211 |
| Non-psychiatric comparisons (vs. Attempters) | 0.11 (0.06) | 1.89 | | .060 | .480 | |
| Age | 0.03 (0.02) | 1.20 | | .232 | 1 | |
| Male sex (vs. Female) | 0.02 (0.05) | 0.49 | | .624 | 1 | |
| **Linear model predicting Conscientiousness** | | | | | | |
| Depressed non-attempter comparisons (vs. Attempters) | -0.09 (0.09) | -1.00 | 280 | .317 | 1 | $R^2$ = 0.20<br>Adj. $R^2$ = 0.19<br>F(4,280) = 17.49<br>*p*-value <.001 |
| Non-psychiatric comparisons (vs. Attempters) | 0.73 (0.10) | 6.95 | | <.001 | <.001 | |
| Age | -0.02 (0.04) | -0.50 | | .615 | 1 | |
| Male sex (vs. Female) | 0.08 (0.08) | 0.95 | | .342 | 1 | |
| **Linear model predicting Agreeableness** | | | | | | |
| Depressed non-attempter comparisons (vs. Attempters) | 0.05 (0.04) | 1.12 | 280 | .263 | 1 | $R^2$ = 0.08<br>Adj. $R^2$ = 0.07<br>F(4,280) = 6.34<br>*p*-value < .001 |
| Non-psychiatric comparisons (vs. Attempters) | 0.17 (0.05) | 3.27 | | .001 | .008 | |
| Age | 0.03 (0.02) | 1.31 | | .192 | 1 | |
| Male sex (vs. Female) | -0.15 (0.04) | -3.80 | | <.001 | <.001 | |

| | | | | | | |
|---|---|---|---|---|---|---|
| **Linear model predicting Grandiose Narcissism** | | | | | | |
| Depressed non-attempter comparisons (vs. Attempters) | 0.04 (0.12) | 0.31 | 280 | .760 | 1 | $R^2$ = 0.08 |
| Non-psychiatric comparisons (vs. Attempters) | -0.38 (0.14) | -2.67 | | .008 | .064 | Adj. $R^2$ = 0.07 |
| Age | 0.01 (0.05) | 0.18 | | .854 | 1 | F(4,280) = 6.31 |
| Male sex (vs. Female) | 0.44 (0.11) | 4.02 | | <.001 | <.001 | *p*-value <.001 |
| **Linear model predicting Need for Closure** | | | | | | |
| Depressed non-attempter comparisons (vs. Attempters) | -0.22 (0.10) | -2.18 | 280 | .030 | .240 | $R^2$ = 0.04 |
| Non-psychiatric comparisons (vs. Attempters) | -0.40 (0.12) | -3.42 | | .001 | .008 | Adj. $R^2$ = 0.03 |
| Age | -0.01 (0.04) | -0.17 | | .869 | 1 | F(4,280) = 3.15 |
| Male sex (vs. Female) | -0.02 (0.09) | -0.24 | | .811 | 1 | *p*-value =.015 |
| **Linear model predicting Borderline traits** | | | | | | |
| Depressed non-attempter comparisons (vs. Attempters) | -5.58 (1.45) | -3.84 | 279 | <.001 | <.001 | $R^2$ = 0.43 |
| Non-psychiatric comparisons (vs. Attempters) | -23.67 (1.69) | -14.04 | | <.001 | <.001 | Adj. $R^2$ = 0.42 |
| Age | 0.35 (0.64) | 0.55 | | .580 | 1 | F(4,279) = 52.03 |
| Male sex (vs. Female) | -1.77 (1.30) | -1.37 | | .172 | 1 | *p*-value <.001 |

*Note*. Results of eight linear regression models predicting personality traits with study groups (reference group: suicide attempters), covarying for mean-centered age and for sex (reference group: female). The t-statistics evaluating the significance of each regression coefficient was obtained based on 280 residual degrees of freedom (285 total observations – 4 predictors – 1) except for the model predicting borderline traits, as one participant was missing data on borderline traits resulting in 284 total observations instead of 285 for this test. The "Adjusted *p*-value" column presents *p*-values adjusted with Bonferroni for eight tests.

**Supplemental Table S3**

*Summary table of considerations underlying the selection of personality traits for the LPA based on their (a) relevance for distinct subpopulations of older attempters, (b) lack of redundance (absence of strong intercorrelations), and (c) lack of differentiation of attempters as a single group from depressed and non-psychiatric comparisons.*

| **Personality traits** | **(a) Evidence in favor of (+) or against (-) associations with distinct suicidal behavior or attempter characteristics in later life** | **(b) Any strong correlations between traits** (Supplemental Figure S1) | **(c) Differentiating attempters as a group from depressed non attempters** (Supplemental Table S2) | **Final decision to include in LPA** | **Expected associations with specific suicidal behavior or attempter characteristics** |
|---|---|---|---|---|---|
| Neuroticism | **(+)** Higher in early-onset attempters aged 50+ years than in depressed non-attempters (Szücs et al., 2020) → in turn, early-onset suicidal behavior has been associated with childhood trauma (Chang et al., 2024), early-onset affective disorders (Gili et al., 2019), substance use disorder (Rioux et al., 2021), and higher impulsivity (Ojala et al., 2022).<br>**(+)** Neuroticism facets have been positively associated with more attempts; lower attempt lethality & intent (Seidlitz et al., 2001) | Strongly correlated with borderline traits (r=0.72) | No | Yes | Higher neuroticism<br>↔<br>More attempts at baseline or reattempt during follow up; lower suicidal intent; lower suicide lethality; earlier age of onset of suicidal behavior; attempters with suicide risk factors for onset of suicidal behavior at a young age (e.g., childhood trauma, , earlier onset of the first depressive episode, substance use disorder, higher impulsivity) |
| Extraversion | **(+)** Higher in late-onset attempters aged 50+ years & depressed non-attempters than early-onset attempters (Szücs et al., 2020)<br>**(+)** Positively associated with fewer attempts in older adults (P. R. Duberstein et al., 2000), also on the facet level (Seidlitz et al., 2001; Useda et al., 2004)<br>**(+)** Qualitative description of older individuals who died by suicide as being cold and distant (Kjølseth et al., 2009)<br>**(+)** Positively associated with tie strength and in social network and larger social network size (Iveniuk, 2019) → social connectedness found to be protective against suicidal ideation in old age (for s review: Ki et al., 2024) | None | No | Yes | Lower extraversion<br>(introversion, coldness, reserve)<br>↔<br>More attempts at baseline or reattempt during follow up; earlier age of onset of suicidal behavior (see associated factors listed under neuroticism); higher attempt lethality (was described in lethal attempts); attempters with low social connectedness (especially low social network diversity and size) and possibly high suicidal ideation |
| Openness to experience | **(+)** Some openness facets have been associated with better cognitive functioning in older adults with early-onset depression, including better cognitive flexibility (Armstrong et al., 2024) → Global cognition and memory deficits were associated with late-onset suicidal behavior (Erlangsen et al., 2008; Gujral et al., 2021); cognitive control deficits (executive dysfunction) associated with high-lethality suicidal behavior in old age (McGirr et al., 2012)<br>**(+)** Lower openness found in older suicide decedents compared to non-suicidal comparisons (Draper 2014; Duberstein et al., | None | No | Yes | Lower openness<br>(closemindedness, lack of curiosity)<br>↔<br>attempters with more cognitive deficits (poorer general cognition; higher executive dysfunction) and potentially later onset and higher lethality suicidal behavior; attempters with some indicator of aging-related decline (lower physical, cognitive, or social health; declining health over time) |

| | | | | | |
|---|---|---|---|---|---|
| | 1994) and theorized to increase maladaptive response to aging-related stressors (Duberstein, 1995) | | | | |
| Agreeableness | **(+)** Qualitative description of older individuals who died by suicide as being authoritarian, with difficulty to show positive feelings towards others (Kjølseth et al., 2009)<br>**(+)** Positively associated with strength of social ties (Iveniuk, 2019) → social connectedness found to be protective against suicidal ideation in old age (for a review: Ki et al., 2024) | None | No | Yes | <u>Lower</u> agreeableness (callousness, antagonism)<br>↔<br>higher attempt lethality (was described in lethal attempts); attempters with low social connectedness (especially low embedded network) and possibly high suicidal ideation |
| Conscientiousness | **(+)** Higher in death by suicide at an older vs younger age (De Leo et al., 2013; Draper et al., 2014)<br>**(+)** Higher likelihood of recent suicidal behavior (≤ 2 years) in older adults and higher suicidal intent in older attempters (Szücs et al., 2023) | None | No | Yes | <u>Higher</u> conscientiousness<br>↔<br>Older age at most recent attempt; higher suicidal intent |
| Grandiose narcissism | **(+)** Linked to suicidal crises during aging in the psychoanalytic literature (Kernberg, 2014; Ronningstam & Maltsberger, 1998)<br>**(+)** Associations of grandiose narcissism with multiple attempts, high suicidal intent, and low suicidal ideation severity found by a systematic review, although evidence in older adults remains scarce (Sprio et al., 2024) | None | No | Yes | <u>Higher</u> levels of grandiose narcissism<br>↔<br>More attempts at baseline or reattempt during follow up; high suicidal intent; low suicidal ideation severity; attempters with some indicator of aging-related decline (lower physical, cognitive, or social health; declining health over time) |
| Need for Closure | **(+)** described in the context of death by suicide in old age in qualitative studies as stubbornness, rigidity (Conwell et al., 1990; Kjolseth et al., 2009), in particular in individuals who experienced functional decline or a high suspicion of a cancer (Conwell et al., 1990; Kjølseth et al., 2010).<br>**(+)** strongly associated with orderliness, a conscientiousness component (Costantini & Perugini, 2016), which differentiated late-onset attempters aged 50+ years from depressed non-attempters, while conscientiousness did not (Szücs et al., 2020). | None | No | Yes | <u>Higher</u> need for closure<br>↔<br>Higher lethality (was described in lethal attempts); later age of onset of suicidal behavior; attempters with some indicator of aging-related decline (lower physical, cognitive, or social health; declining health over time) |
| Borderline personality | **(+)** Higher in early-onset attempters aged 50+ years than in depressed non-attempters (Szücs et al., 2020)<br>**(-)** Found to differentiate suicide attempters from non-attempters across the lifespan (Buerke et al., 2021) → general risk factor for suicidal behavior; less likely to characterize specific older subpopulations of attempters.<br>**(-)** Mostly characterizing general personality pathology (Hopwood et al., 2011; Sharp et al., 2015), with none to few specific elements except for instability (Hopwood et al., 2011), which is likely also captured by neuroticism, as in our prior study (Szücs et al., 2020). | Strongly correlated with Neuroticism (r=0.72) | Yes (→ more likely to characterize attempters as a whole than other traits) | No | <u>Higher</u> borderline traits<br>↔<br>The expected associations are the same ones as with high neuroticism |

*Note*. For each personality trait, the last column indicates expectations of associations with specific suicidal behavior characteristics (dark grey highlights) and attempter characteristics (light grey highlights) based on extant direct and indirect evidence (articles listed below).

<u>References for Supplemental Table S3</u>:

Armstrong, M., Kaufman, J., Maciarz, J., Sullivan, D., Kim, J., Koppelmans, V., Langenecker, S., & Weisenbach, S. L. (2024). The relationship between personality and cognition in older adults with and without early-onset depression. *Frontiers in Psychiatry*, *15*. https://doi.org/10.3389/fpsyt.2024.1337320

Buerke, M., Galfalvy, H., Keilp, J. G., Sheftall, A. H., Burke, A. K., Bridge, J. A., Mann, J. J., & Szanto, K. (2021). Age effects on clinical and neurocognitive risk factors for suicide attempt in depression—Findings from the AFSP lifespan study. *Journal of Affective Disorders*, *295*, 123–130. https://doi.org/10.1016/j.jad.2021.08.014

Chang, Y.-W., Buerke, M., Galfalvy, H., & Szanto, K. (2024). Childhood trauma is associated with early-onset but not late-onset suicidal behavior in late-life depression. *International Psychogeriatrics*, *36*(5), 371–384. https://doi.org/10.1017/S1041610223000662

Conwell, Y., Caine, E. D., & Olsen, K. (1990). Suicide and cancer in late life. *Hospital & Community Psychiatry*, *41*(12), 1334–1339.

Costantini, G., & Perugini, M. (2016). The network of conscientiousness. *Journal of Research in Personality*, *65*, 68–88. https://doi.org/10.1016/j.jrp.2016.10.003

De Leo, D., Draper, B. M., Snowdon, J., & Kõlves, K. (2013). Suicides in older adults: A case-control psychological autopsy study in Australia. *Journal of Psychiatric Research*, *47*(7), Article 7. https://doi.org/10.1016/j.jpsychires.2013.02.009

Draper, B., Kõlves, K., De Leo, D., & Snowdon, J. (2014). A controlled study of suicide in middle-aged and older people: Personality traits, age, and psychiatric disorders. *Suicide & Life-Threatening Behavior*, *44*(2), Article 2. https://doi.org/10.1111/sltb.12053

Duberstein, P. R., Conwell, Y., & Caine, E. D. (1994). Age differences in the personality characteristics of suicide completers: Preliminary findings from a psychological autopsy study. Psychiatry, 57(3), 213–224.

Duberstein, P. (1995). Openness to Experience and Completed Suicide Across the Second Half of Life. *International Psychogeriatrics*, *7*(2), Article 2. https://doi.org/10.1017/S1041610295001967

Duberstein, P. R., Conwell, Y., Seidlitz, L., Denning, D. G., Cox, C., & Caine, E. D. (2000). Personality traits and suicidal behavior and ideation in depressed inpatients 50 years of age and older. *Journals of Gerontology Series B-Psychological Sciences & Social Sciences*, *55*(1), Article 1.

Erlangsen, A., Zarit, S. H., & Conwell, Y. (2008). Hospital-Diagnosed Dementia and Suicide: A Longitudinal Study Using Prospective, Nationwide Register Data. *American Journal of Geriatric Psychiatry*, *16*(3), Article 3. https://doi.org/10.1097/JGP.0b013e3181602a12

Gili, M., Castellví, P., Vives, M., de la Torre-Luque, A., Almenara, J., Blasco, M. J., Cebrià, A. I., Gabilondo, A., Pérez-Ara, M. A., A, M.-M., Lagares, C., Parés-Badell, O., Piqueras, J. A., Rodríguez-Jiménez, T., Rodríguez-Marín, J., Soto-Sanz, V., Alonso, J., & Roca, M. (2019). Mental disorders as risk factors for suicidal behavior in young people: A meta-analysis and systematic review of longitudinal studies. *Journal of Affective Disorders*, *245*, 152–162. https://doi.org/10.1016/j.jad.2018.10.115

Gujral, S., Butters, M. A., Dombrovski, A. Y., & Szanto, K. (2021). Late-Onset Suicide: A Dementia Prodrome? *The American Journal of Geriatric Psychiatry*, *29*(7), 709–713. https://doi.org/10.1016/j.jagp.2020.12.004

Hopwood, C. J., Malone, J. C., Ansell, E. B., Sanislow, C. A., Grilo, C. M., McGlashan, T. H., Pinto, A., Markowitz, J. C., Shea, M. T., Skodol, A. E., Gunderson, J. G., Zanarini, M. C., & Morey, L. C. (2011, July 1). *Personality Assessment in DSM-5: Empirical Support for Rating Severity, Style, and Traits* (world) [Research-article]. Https://Doi.Org/10.1521/Pedi.2011.25.3.305; Guilford Publications Inc. https://doi.org/10.1521/pedi.2011.25.3.305

Iveniuk, J. (2019). Social Networks, Role-Relationships, and Personality in Older Adulthood. *The Journals of Gerontology: Series B*, *74*(5), 815–826. https://doi.org/10.1093/geronb/gbx120

Kernberg, O. F. (2014). An overview of the treatment of severe narcissistic pathology. *The International Journal of Psychoanalysis*, *95*(5), 865–888. https://doi.org/10.1111/1745-8315.12204

Ki, M., Lapierre, S., Gim, B., Hwang, M., Kang, M., Dargis, L., Jung, M., Koh, E. J., & Mishara, B. (2024). A systematic review of psychosocial protective factors against suicide and suicidality among older adults. *International Psychogeriatrics*, *36*(5), 346–370. https://doi.org/10.1017/S104161022300443X

Kjølseth, I., Ekeberg, O., & Steihaug, S. (2009). “Why do they become vulnerable when faced with the challenges of old age?” Elderly people who committed suicide, described by those who knew them. *International Psychogeriatrics*, *21*(5), Article 5. https://doi.org/10.1017/S1041610209990342

Kjolseth, I., Ekeberg, O., & Steihaug, S. (2009). “Why do they become vulnerable when faced with the challenges of old age?” Elderly people who committed suicide, described by those who knew them. *International Psychogeriatrics*, *21*(5), 903–912. https://doi.org/10.1017/S1041610209990342

Kjølseth, I., Ekeberg, Ø., & Steihaug, S. (2010). Why suicide? Elderly people who committed suicide and their experience of life in the period before their death. *International Psychogeriatrics*, *22*(02), 209. https://doi.org/10.1017/S1041610209990949

McGirr, A., Dombrovski, A. Y., Butters, M., Clark, L., & Szanto, K. (2012). Deterministic learning and attempted suicide among older depressed individuals: Cognitive assessment using the Wisconsin Card Sorting Task. *Journal of Psychiatric Research*, *46*(2), Article 2. https://doi.org/10.1016/j.jpsychires.2011.10.001

Ojala, O., Kuja-Halkola, R., Bjureberg, J., Ohlis, A., Cederlöf, M., Norén Selinus, E., Lichtenstein, P., Larsson, H., Lundström, S., & Hellner, C. (2022). Associations of impulsivity, hyperactivity, and inattention with nonsuicidal self-injury and suicidal behavior: Longitudinal cohort study following children at risk for neurodevelopmental disorders into mid-adolescence. *BMC Psychiatry*, *22*(1), 679. https://doi.org/10.1186/s12888-022-04311-5

Rioux, C., Huet, A.-S., Castellanos-Ryan, N., Fortier, L., Blanc, M. L., Hamaoui, S., Geoffroy, M.-C., Renaud, J., & Séguin, J. R. (2021). Substance use disorders and suicidality in youth: A systematic review and meta-analysis with a focus on the direction of the association. *PLOS ONE*, *16*(8), e0255799. https://doi.org/10.1371/journal.pone.0255799

Ronningstam, E. F., & Maltsberger, J. T. (1998). Pathological narcissism and sudden suicide-related collapse. *Suicide and Life-Threatening Behavior*, *28*(3), Article 3.

Seidlitz, L., Conwell, Y., Duberstein, P., Cox, C., & Denning, D. (2001). Emotion traits in older suicide attempters and non-attempters. *J Affect Disord*, *66*(2–3), Article 2–3.

Sharp, C., Wright, A. G., Fowler, J. C., Frueh, B. C., Allen, J. G., Oldham, J., & Clark, L. A. (2015). The structure of personality pathology: Both general (‘g’) and specific (‘s’) factors? *Journal of Abnormal Psychology*, *124*(2), Article 2.

Sprio, V., Mirra, L., Madeddu, F., Lopez-Castroman, J., Blasco-Fontecilla, H., Di Pierro, R., & Calati, R. (2024). Can clinical and subclinical forms of narcissism be considered risk factors for suicide-related outcomes? A systematic review. *Journal of Psychiatric Research*, *172*, 307–333. https://doi.org/10.1016/j.jpsychires.2024.02.017

Szücs, A., Galfalvy, H. C., Alessi, M. G., Kenneally, L. B., Valderas, J. M., Maier, A. B., & Szanto, K. (2023). Diligent for Better or Worse: Trait Conscientiousness in Suicidal Ideation and Behavior and Its Moderating Effect on Ageing [Poster Presentation]. *Society of Biological Psychiatry, San Diego, CA, United States.*

Szücs, A., Szanto, K., Wright, A. G., & Dombrovski, A. Y. (2020). Personality of late-and early-onset elderly suicide attempters. *International Journal of Geriatric Psychiatry*, *35*(4), 384–395.

Useda, J. D., Duberstein, P. R., Conner, K. R., & Conwell, Y. (2004). Personality and attempted suicide in depressed adults 50 years of age and older: A facet level analysis. *Compr Psychiatry*, *45*(5), Article 5.

**Supplemental Table S4**

*Goodness-of-fit statistics for LPA solutions with three profiles*

| | AIC | BIC | Entropy | Minimum Posterior Probability | Maximum Posterior Probability | BLRT - *p*-value |
|---|---|---|---|---|---|---|
| **equal variances, zero covariances** | 2127.87 | 2208.61 | 0.74 | 0.82 | 0.92 | 0.0099 |
| **varying variances, zero covariances** | 2139.62 | 2258.04 | 0.79 | 0.88 | 0.92 | 0.0099 |
| **equal variances, equal covariances** | 2092.80 | 2230.06 | 0.86 | 0.92 | 0.97 | 0.0495 |
| **varying variances, varying covariances** | 2117.00 | 2404.98 | 0.89 | 0.95 | 0.96 | 0.2673 |

*Note*. Legend: BLRT, Bootstrapped Likelihood Ratio Test

## Supplemental Figure S2

*K-means cluster solutions*

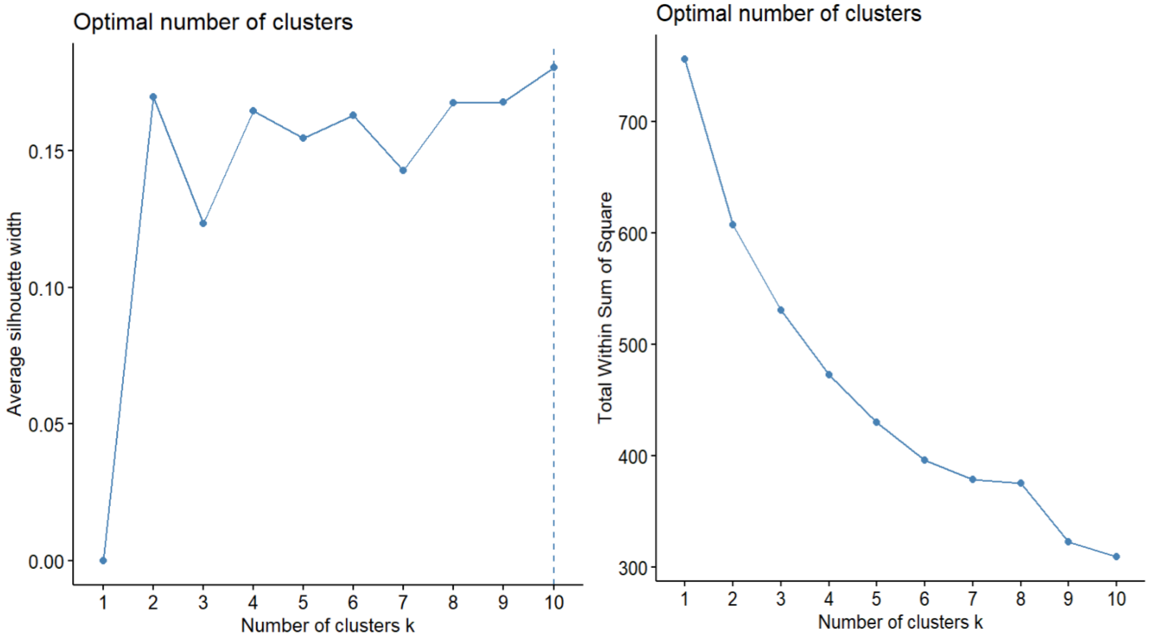


## Supplemental Table S5

*K-means cluster characteristics*

| | Cluster 1 [C1] | Cluster 2 [C2] | Cluster 3 [C3] | F-statistic; Cohen's f | *p*-value | Tukey-adjusted pairwise contrasts |
|---|---|---|---|---|---|---|
| **n** | 47 | 24 | 38 | | | |
| **Jaccard index** | 0.74 | 0.75 | 0.62 | | | |
| **Stability index** | 0.86 | 0.84 | 0.64 | | | |
| **Mean scores of personality measures used for clustering** | | | | | | |
| **Neuroticism** | 4.03 (0.55) | 3.02 (0.82) | 2.77 (0.65) | 43.59; 0.91 | **<.001** | C2,C3<C1 |
| **Extraversion** | 2.45 (0.54) | 3.16 (0.58) | 3.27 (0.56) | 26.84; 0.71 | **<.001** | C1<C2,C3 |
| **Openness to experience** | 3.16 (0.39) | 3.27 (0.29) | 3.12 (0.34) | 1.36; 0.16 | .261 | - |
| **Agreeableness** | 3.97 (0.25) | 3.41 (0.27) | 3.98 (0.32) | 37.61; 0.84 | **<.001** | C3<C1,C2 |
| **Conscientiousness** | 2.94 (0.65) | 3.62 (0.61) | 3.91 (0.58) | 27.39; 0.72 | **<.001** | C1<C2,C3 |
| **Grandiose narcissism** | 1.89 (0.70) | 2.99 (0.66) | 1.09 (0.69) | 56.82; 1.04 | **<.001** | C2<C1<C3 |
| **Need for Closure** | 4.04 (0.73) | 4.11 (0.73) | 4.09 (0.81) | 0.09; 0.04 | .917 | - |

**Supplemental Table S6**

*Descriptive statistics of age at the first attempt, age at the most recent attempt, and age at baseline in attempters*

| Age at first attempt (years) | Age at most recent attempt (years) | Age at baseline in attempters (years) |
|---|---|---|
| 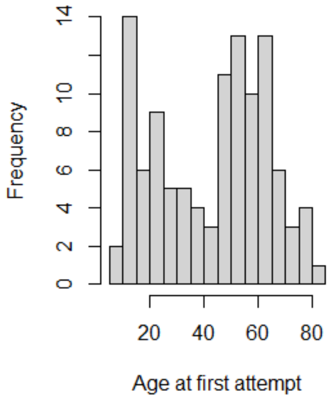 | 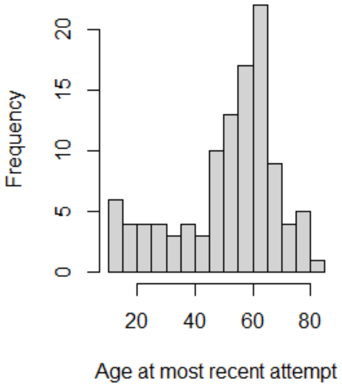 | 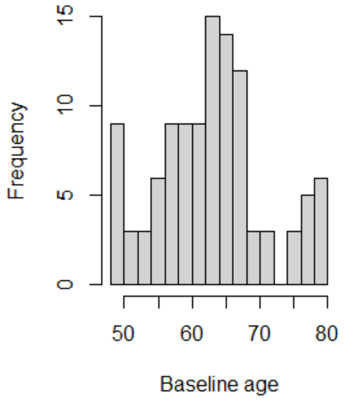 |
| Mean = 43.32<br>SD = 20.72<br>Median = 48<br>Min-Max = [7:81]<br>Skewedness = -0.17<br>Kurtosis = -1.28 | Mean = 51.64<br>SD = 17.55<br>Median = 56<br>Min-Max = [11:81]<br>Skewedness = -0.8<br>Kurtosis = -0.2 | Mean = 63.08<br>SD = 7.91<br>Median = 63<br>Min-Max = [48:79]<br>Skewedness = 0.2<br>Kurtosis = -0.34 |

## Supplemental Table S7

*All cross-sectional group comparisons on baseline individual characteristics*

| **Total N = 285**<br>*Variables also presented in the main article are **bolded*** | **Careless Labile Attempters [CL]**<br>n = 71<br>means (SD) | **Callous Narcissistic Attempters [CN]**<br>n = 25<br>mean (SD) | **Rigid Extraverted Attempters [RE]**<br>n = 13<br>mean (SD) | **Depressed Non-Attempter Comparisons [DNA]**<br>n = 111<br>mean (SD) | **Non-Psychiatric Comparisons [NPC]**<br>n = 65<br>mean (SD) | **Test statistic**<br>*F-statistic unless other test specified* | **Degrees of freedom** | **Effect size**<br>*Cohen's f unless otherwise specified* | **Adjusted *p*-value**<br>*Benjamini-Hochberg correction for 29 tests* | **Post hoc pairwise contrasts**<br>*Adjusted with Tukey unless otherwise specified* |
|---|---|---|---|---|---|---|---|---|---|---|
| **Personality measures used in the LPA** | | | | | | | | | | |
| **Neuroticism (NEO-FFI)** | 3.70 (0.79) | 2.90 (0.63) | 2.40 (0.59) | 3.23 (0.68) | 1.77(0.43) | 85.22 | 4, 280 | 1.10 | <.001 | NPC<other groups<CL<br>RE<DNA |
| **Extraversion (NEO-FFI)** | 2.71 (0.61) | 3.02 (0.58) | 3.62 (0.67) | 2.84 (0.60) | 3.46 (0.50) | 20.08 | 4, 280 | 0.54 | <.001 | CL,CN,DNA<RE,NPC |
| Openness to experience (NEO-FFI) | 3.19 (0.35) | 3.14 (0.37) | 3.10 (0.33) | 3.19 (0.37) | 3.29 (0.42) | 1.26 | 4, 280 | 0.13 | 1 | - |
| **Agreeableness (NEO-FFI)** | 3.95 (0.28) | 3.40 (0.24) | 4.19 (0.20) | 3.89 (0.34) | 4.01 (0.28) | 22.89 | 4, 280 | 0.57 | <.001 | CN<other groups<br>DNA<RE |
| **Conscientiousness (NEO-FFI)** | 3.27 (0.78) | 3.68 (0.62) | 3.86 (0.54) | 3.35 (0.68) | 4.16 (0.43) | 21.42 | 4, 280 | 0.55 | <.001 | CL<RE, NPC<br>CN,DNA<NPC |
| **Grandiose narcissism (BPNI-G)** | 1.61 (0.81) | 2.78 (0.80) | 1.38 (1.06) | 1.92 (0.93) | 1.51 (0.85) | 11.62 | 4, 280 | 0.41 | <.001 | other groups<CN<br>NPC<DNA |
| Need for closure (NFC) | 3.98 (0.73) | 3.91 (0.69) | 4.88 (0.54) | 3.85 (0.75) | 3.67 (0.75) | 8.09 | 4, 280 | 0.34 | <.001 | other groups<RE |
| **Other personality measure** | | | | | | | | | | |
| **Borderline traits (PAI-BOR); n=284** | 34.85 (12.69) | 31.80 (15.80) | 20.00 (11.55) | 26.68 (9.42) | 8.40 (4.73) | 60.92 | 4, 279 | 0.93 | <.001 | RE, DNA<CL<br>RE<CN<br>NPC<other groups |
| **Socio-demographics** | | | | | | | | | | |
| Age | 59.79 (7.78) | 61.88 (7.10) | 66.15 (9.03) | 62.93 (6.79) | 63.75 (7.28) | 0.80 | 4, 280 | 0.11 | 1 | - |
| Female sex - count (%) | 51 (71.83) | 10 (40.00) | 7 (53.85) | 101 (56.11) | 76 (54.29) | 9.66[a] | 4 | 0.14[d] | 1 | - |
| Race - count (%) | | | | | | [b] | 4 | - | .118 | - |

| **Total N = 285**<br>*Variables also presented in the main article are **bolded*** | **Careless Labile Attempters [CL]**<br>n = 71<br>means (SD) | **Callous Narcissistic Attempters [CN]**<br>n = 25<br>mean (SD) | **Rigid Extraverted Attempters [RE]**<br>n = 13<br>mean (SD) | **Depressed Non-Attempter Comparisons [DNA]**<br>n = 111<br>mean (SD) | **Non-Psychiatric Comparisons [NPC]**<br>n = 65<br>mean (SD) | **Test statistic**<br>*F-statistic unless other test specified* | **Degrees of freedom** | **Effect size**<br>*Cohen's f unless otherwise specified* | **Adjusted *p*-value**<br>*Benjamini-Hochberg correction for 29 tests* | **Post hoc pairwise contrasts**<br>*Adjusted with Tukey unless otherwise specified* |
|---|---|---|---|---|---|---|---|---|---|---|
| *White* | 57 (80.28%) | 19 (76.00%) | 11 (84.62%) | 95 (85.59%) | 62 (95.38%) | | | | | |
| *African-American* | 11 (15.49%) | 5 (20.00%) | 2 (15.38%) | 15 (13.51%) | 3 (4.62%) | | | | | |
| *Asian* | 1 (1.41%) | 1 (4.00%) | 0 (0.00%) | 1 (0.90%) | 0 (0.00%) | | | | | |
| *More than one race* | 2 (2.82%) | 0 (0.00%) | 0 (0.00%) | 0 (0.00%) | 0 (0.00%) | | | | | |
| Education in years | 14.64 (2.87) | 13.32 (2.73) | 13.31 (2.87) | 15.59 (2.66) | 16.45 (2.46) | 9.48 | 4, 280 | 0.37 | <.001 | CL, RE, CN<NPC<br>CN, RE<DNA |
| **Per Capita Yearly Income; n=274** | 19,927 (12,463) | 17,859 (11,347) | 19,965 (11,870) | 25,227 (19,713) | 39,527 (17,548) | 14.22 | 4, 269 | 0.46 | <.001 | other groups<NPC |
| **Mental health** | | | | | | | | | | |
| Depression (HDRS); n=220 | 20.48 (5.46) | 19.24 (5.87) | 21.69 (6.68) | 17.62 (4.60) | - | 5.76 | 3, 216 | 0.28 | .006 | DNA<CL, RE |
| Age of onset of first depressive episode; n=195 | 31.10 (20.29) | 30.10 (17.37) | 49.50 (20.61) | 36.54 (17.39) | - | 4.08 | 3, 191 | 0.25 | .210 | - |
| **Worst suicidal ideation (SSI) - median [IQR]; n=220** | 7 [1-21] | 11 [1-21] | 0 [0-21] | 0 [0-1] | - | 60.48[c] | 3 | 0.27[e] | <.001 | DNA<CL, CN[f] |
| Lifetime substance use disorder (SCID) – count (%); n=214 | 40 (57.14%) | 16 (69.57%) | 7 (53.85%) | 50 (46.30%) | - | 4.96[a] | 3 | 0.10[d] | 1 | - |
| Baseline substance use disorder (SCID) – count (%); n=214 | 12 (17.14%) | 2 (8.70%) | 5 (38.46%) | 8 (7.41%) | - | [b] | 3 | - | .291 | - |
| **Cognitive health** | | | | | | | | | | |
| Cognitive functioning – brief assessment (MMSE); n = 278 | 28.37 (1.53) | 27.64 (2.42) | 27.85 (1.63) | 29.01(1.06) | 29.19 (1.03) | 9.18 | 4, 278 | 0.37 | <.001 | attempter groups<DNA,NPC |
| **Cognitive functioning – detailed assessment (DRS); n=262** | 136.11 (4.01) | 133.50 (4.83) | 132.08 (6.37) | 136.78 (4.13) | 138.97 (2.71) | 12.53 | 4, 257 | 0.44 | <.001 | other groups < NPC<br>RE, CN < DNA<br>RE < CL |
| Executive functioning (EXIT); n=253 | 7.16 (3.22) | 8.68 (3.77) | 7.36 (4.92) | 6.61 (3.62) | 5.20 (2.43) | 5.40 | 4, 248 | 0.30 | .010 | NPC<CL,CN |

| **Total N = 285** *Variables also presented in the main article are **bolded*** | **Careless Labile Attempters [CL]** n = 71 means (SD) | **Callous Narcissistic Attempters [CN]** n = 25 mean (SD) | **Rigid Extraverted Attempters [RE]** n = 13 mean (SD) | **Depressed Non-Attempter Comparisons [DNA]** n = 111 mean (SD) | **Non-Psychiatric Comparisons [NPC]** n = 65 mean (SD) | **Test statistic** *F-statistic unless other test specified* | **Degrees of freedom** | **Effect size** *Cohen's f unless otherwise specified* | **Adjusted *p*-value** *Benjamini-Hochberg correction for 29 tests* | **Post hoc pairwise contrasts** *Adjusted with Tukey unless otherwise specified* |
|---|---|---|---|---|---|---|---|---|---|---|
| **Impulsivity (BIS); n=281** | 70.06 (11.43) | 70.08 (12.76) | 67.54 (12.68) | 65.65 (9.48) | 51.89 (7.64) | 33.02 | 4, 276 | 0.69 | <.001 | DNA<CL NPC<other groups |
| **Anger rumination (ARS); n=284** | 38.33 (10.86) | 39.20 (11.54) | 29.62 (7.03) | 35.60 (9.14) | 24.48 (4.70) | 26.00 | 4, 279 | 0.61 | <.001 | RE,NPC< CL,CN NPC<DNA |
| **Physical health** | | | | | | | | | | |
| **Physical illness burden (CIRS-G) – median [IQR]; n=270** | 9 [5-13] | 8 [5.25-11.75] | 9 [6-14.5] | 9 [6-13] | 4.5 [2-6] | 46.88[c] | 4 | 0.16[e] | <.001 | NPC<other groups[f] |
| CIRS-G Severe physical illness (score of 3 or 4 in any organ system) – count (%); n=270 | 25 (35.21%) | 7 (28.00%) | 3 (23.08%) | 28 (25.22%) | 5 (7.69%) | [b] | 4 | - | .062 | - |
| **Social health** | | | | | | | | | | |
| **SNI – Network diversity – median [IQR]; n=284** | 3 [2-5] | 4 [2-5] | 5 [4-5] | 4 [3-6] | 6 [5-7] | 45.31[c] | 4 | 0.15[e] | <.001 | CL,CN,DNA<NPC[f] |
| SNI – Number of people – median [IQR]; n=284 | 7 [3.25-11] | 8 [4-9] | 9 [7-15] | 9 [4-13.5] | 13 [9-19] | 35.94[c] | 4 | 0.11[e] | <.001 | CL,CN,DNA<NPC[f] |
| SNI – Embedded network – median [IQR]; n=284 | 0 [0-1] | 0 [0-1] | 1 [1-1] | 1 [0-2] | 1 [1-2] | 28.96[c] | 4 | 0.09[e] | <.001 | CL,CN,DNA<NPC[f] |
| **Childhood Trauma (CTQ) – median [IQR]; n=269** | 51 [37-63.5] | 48 [35.5-65] | 33.5 [26.75-49.25] | 44.5 [33.75-52.25] | 28 [25-33] | 74.86[c] | 4 | 0.27[e] | <.001 | NPC<CL,CN,DNA; RE<CL[f] |

*Note.* Dark grey columns indicate attempter profiles identified by LPA; light grey columns indicate non-attempter comparison groups. Values in all grey columns represent mean (standard deviation) unless otherwise specified. Differences having a large effect size (Cohen's f ≥ 0.40 or $\eta^2 \geq 0.14$ or Cramer's V ≥ 0.6) are bolded. P-values have been adjusted for 29 tests using Benjamini-Hochberg's method. The number of observations is specified under variables with missing data. Legend: IQR, interquartile range; NEO-FFI, NEO Five-Factor Inventory; BPNI-G, Brief Pathological Narcissism Inventory – Grandiosity subscale; PAI-BOR, Personality Assessment Inventory-Borderline Scale; HDRS, Hamilton Rating Scale for Depression; SSI; Beck Scale of Suicidal Ideation; SCID, Structured Clinical Interview for DSM-IV disorders; MMSE, Mini Mental State Exam; DRS, Global Cognitive Mattis Dementia Rating Scale; BIS, Barratt Impulsivity Scale; ARS, Anger Rumination Scale; CIRS-G, Cumulative Illness Rating Scale – Geriatrics; SNI, Social Network Index; CTQ, Childhood Trauma Questionnaire;

[a], Chi-square test value; [b], Fisher's exact test (this test has no test statistic); [c], Kruskal Wallis H-statistic; [d], Effect size computed as Cramer's V; [e], Effect size computed as $\eta^2$; [f], Post hoc pairwise contrasts adjusted with Holm's method.

**Supplemental Table S8**

*Adjusted analyses covarying for age and sex of attempter profile differences in suicidal behavior characteristics (n = 109)*

| **Attempter subsample: n = 109** | **Careless Labile Attempters [CL] n = 71 65.14%** | **Callous Narcissistic Attempters [CN] n = 25 22.94%** | **Rigid Extraverted Attempters [RE] n = 13 11.92%** | **Test statistic** *F-statistic unless otherwise specified* | **Degrees of freedom** | **Effect size** *Cohen's f unless otherwise specified* | **Adjusted *p*-value** *Benjamini-Hochberg correction for 6 tests* | **Tukey-adjusted post hoc pairwise contrasts** |
|---|---|---|---|---|---|---|---|---|
| **Suicidal behavior characteristics** (tests adjusted for age and sex) | | | | | | | | |
| Maximum lethality of suicidal behavior (BLS); n=106 | 3.28 (1.81) | 3.50 (2.32) | 4.08 (2.40) | 0.87 | 2, 101 | 0.13 | 1 | - |
| Intent score of the most lethal suicide attempt (SIS); n=106 | 17.96 (5.02) | 20.96 (4.39) | 17.08 (3.75) | 4.40 | 2, 101 | 0.30 | .088 | - |
| **Planning score for the most lethal suicide attempt (SIS Planning subscale); n=107** | 7.64 (2.88) | 9.40 (3.10) | 5.69 (1.60) | 7.78 | 2, 102 | 0.39 | .004 | CL,RE<CN |
| Number of lifetime attempts – median [IQR] | 1 [1-3] | 1 [1-3] | 1 [1-2] | 3.18[a] | 2, 104 | 0.17[b] | 1 | - |
| **Age at first attempt in years** | 40.23 (20.52) | 43.24(18.86) | 60.38 (18.01) | 6.03 | 2, 104 | 0.34 | .020 | CL,CN<RE |
| Age at most recent attempt in years | 49.15 (17.79) | 52.16 (16.71) | 64.23 (12.66) | 4.30 | 2, 104 | 0.29 | .096 | - |

*Note.* Group differences in adjusted analyses did not differ significantly from group differences in the unadjusted analysis (Table 1). Columns in grey represent mean (SD) for the three attempter profiles unless otherwise specified. The number of observations is specified for variables with missing data. P-values have been adjusted for 6 tests using Benjamini-Hochberg's method. Legend: LPA, latent profile analysis; BLS, Beck Lethality scale, SIS, Beck Suicidal Intent Scale.

[a], Likelihood ratio chi-square statistic obtained from negative binomial logistic regression; [b], Effect size computed as Cramer's V.

## Supplemental Table S9

*Adjusted analyses of cross-sectional group comparisons on baseline individual characteristics* *covarying for age and sex (and for education for cognitive health)*

| **Total N = 285**<br>*Variables also presented in the main article are* ***bolded*** | **Careless Labile Attempters [CL]**<br>n = 71<br>means (SD) | **Callous Narcissistic Attempters [CN]**<br>n = 25<br>mean (SD) | **Rigid Extraverted Attempters [RE]**<br>n = 13<br>mean (SD) | **Depressed Non-Attempter Comparisons [DNA]**<br>n = 111<br>mean (SD) | **Non-Psychiatric Comparisons [NPC]**<br>n = 65<br>mean (SD) | **Test statistic**<br>*F-statistic unless other test specified* | **Degrees of freedom** | **Effect size**<br>*Cohen's f unless otherwise specified* | **Adjusted *p*-value**<br>*Benjamini-Hochberg correction for 29 tests* | **Tukey-adjusted post hoc pairwise contrasts** |
|---|---|---|---|---|---|---|---|---|---|---|
| **Personality measures used in the LPA** (tests adjusted for age and sex) | | | | | | | | | | |
| **Neuroticism (NEO-FFI)** | 3.70 (0.79) | 2.90 (0.63) | 2.40 (0.59) | 3.23 (0.68) | 1.77(0.43) | 84.98 | 4, 278 | 1.11 | <.001 | NPC<other groups<CL<br>RE<DNA |
| **Extraversion (NEO-FFI)** | 2.71 (0.61) | 3.02 (0.58) | 3.62 (0.67) | 2.84 (0.60) | 3.46 (0.50) | 20.09 | 4, 278 | 0.54 | <.001 | CL,CN,DNA<RE,NPC |
| Openness to experience (NEO-FFI) | 3.19 (0.35) | 3.14 (0.37) | 3.10 (0.33) | 3.19 (0.37) | 3.29 (0.42) | 1.26 | 4, 278 | 0.13 | 1 | - |
| **Agreeableness (NEO-FFI)** | 3.95 (0.28) | 3.40 (0.24) | 4.19 (0.20) | 3.89 (0.34) | 4.01 (0.28) | 23.54 | 4, 278 | 0.58 | <.001 | CN<other groups<br>DNA<RE |
| **Conscientiousness (NEO-FFI)** | 3.27 (0.78) | 3.68 (0.62) | 3.86 (0.54) | 3.35 (0.68) | 4.16 (0.43) | 21.28 | 4, 278 | 0.55 | <.001 | CL<RE, NPC<br>CN,DNA<NPC |
| **Grandiose narcissism (BPNI-G)** | 1.61 (0.81) | 2.78 (0.80) | 1.38 (1.06) | 1.92 (0.93) | 1.51 (0.85) | 12.05 | 4, 278 | 0.42 | <.001 | other groups<CN<br>NPC<DNA |
| Need for closure (NFC) | 3.98 (0.73) | 3.91 (0.69) | 4.88 (0.54) | 3.85 (0.75) | 3.67 (0.75) | 8.09 | 4, 278 | 0.34 | <.001 | other groups<RE |
| **Other personality measure** (tests adjusted for age and sex) | | | | | | | | | | |
| **Borderline traits (PAI-BOR); n=284** | 34.85 (12.69) | 31.80 (15.80) | 20.00 (11.55) | 26.68 (9.42) | 8.40 (4.73) | 60.77 | 4, 277 | 0.94 | <.001 | RE, DNA<CL<br>RE<CN<br>NPC<other groups |
| **Socio-demographics** (tests adjusted for age and sex) | | | | | | | | | | |
| Age | 59.79 (7.78) | 61.88 (7.10) | 66.15 (9.03) | 62.93 (6.79) | 63.75 (7.28) | 0.80 | 4, 279 | 0.11 | 1 | - |
| Female sex - count (%) | 51 (71.83) | 10 (40.00) | 7 (53.85) | 101 (56.11) | 76 (54.29) | 9.86[a] | 4, 279 | 0.19[e] | 1 | - |
| Race - count (%) | | | | | | 26.97[b] | 15, 269 | 0.13[e] | 1 | - |

| **Total N = 285**<br><br>*Variables also presented in the main article are **bolded*** | **Careless Labile Attempters [CL]**<br>n = 71<br>means (SD) | **Callous Narcissistic Attempters [CN]**<br>n = 25<br>mean (SD) | **Rigid Extraverted Attempters [RE]**<br>n = 13<br>mean (SD) | **Depressed Non-Attempter Comparisons [DNA]**<br>n = 111<br>mean (SD) | **Non-Psychiatric Comparisons [NPC]**<br>n = 65<br>mean (SD) | **Test statistic**<br><br>*F-statistic unless other test specified* | **Degrees of freedom** | **Effect size**<br><br>*Cohen's f unless otherwise specified* | **Adjusted *p*-value**<br><br>*Benjamini-Hochberg correction for 29 tests* | **Tukey-adjusted post hoc pairwise contrasts** |
|---|---|---|---|---|---|---|---|---|---|---|
| *White* | 57 (80.28%) | 19 (76.00%) | 11 (84.62%) | 95 (85.59%) | 62 (95.38%) | | | | | |
| *African-American* | 11 (15.49%) | 5 (20.00%) | 2 (15.38%) | 15 (13.51%) | 3 (4.62%) | | | | | |
| *Asian* | 1 (1.41%) | 1 (4.00%) | 0 (0.00%) | 1 (0.90%) | 0 (0.00%) | | | | | |
| *More than one race* | 2 (2.82%) | 0 (0.00%) | 0 (0.00%) | 0 (0.00%) | 0 (0.00%) | | | | | |
| Education in years | 14.64 (2.87) | 13.32 (2.73) | 13.31 (2.87) | 15.59 (2.66) | 16.45 (2.46) | 9.68 | 4, 278 | 0.37 | <.001 | CL, RE, CN<NPC<br>CN, RE<DNA |
| **Per Capita Yearly Income; n=274** | 19,927 (12,463) | 17,859 (11,347) | 19,965 (11,870) | 25,227 (19,713) | 39,527 (17,548) | 14.20 | 4, 267 | 0.46 | <.001 | other groups<NPC |
| **Mental health** (tests adjusted for age and sex) | | | | | | | | | | |
| Depression (HDRS); n=220 | 20.48 (5.46) | 19.24 (5.87) | 21.69 (6.68) | 17.62 (4.60) | - | 5.75 | 3, 214 | 0.28 | .025 | DNA<CL, RE |
| Age of onset of first depressive episode; n=195 | 31.10 (20.29) | 30.10 (17.37) | 49.50 (20.61) | 36.54 (17.39) | - | 4.12 | 3, 189 | 0.26 | .215 | - |
| **Worst suicidal ideation (SSI) - median [IQR]; n=220** | 7 [1-21] | 11 [1-21] | 0 [0-21] | 0 [0-1] | - | 41.16[c] | 3, 214 | 0.43[e] | <.001 | DNA<CL, CN |
| Lifetime substance use disorder (SCID) – count (%); n=214 | 40 (57.14%) | 16 (69.57%) | 7 (53.85%) | 50 (46.30%) | - | 5.05[a] | 3, 208 | 0.15[e] | 1 | - |
| Current substance use disorder (SCID) – count (%); n=214 | 12 (17.14%) | 2 (8.70%) | 5 (38.46%) | 8 (7.41%) | - | 10.14[a] | 3, 208 | 0.22[e] | .505 | - |
| **Cognitive health** (tests adjusted for age, sex, and education) | | | | | | | | | | |
| Cognitive functioning – brief assessment (MMSE); n = 278 | 28.37 (1.53) | 27.64 (2.42) | 27.85 (1.63) | 29.01(1.06) | 29.19 (1.03) | 9.70 | 4, 270 | 0.38 | <.001 | attempter groups<DNA,NPC |
| **Cognitive functioning – detailed assessment (DRS); n=262** | 136.11 (4.01) | 133.50 (4.83) | 132.08 (6.37) | 136.78 (4.13) | 138.97 (2.71) | 13.74 | 4, 254 | 0.47 | <.001 | other groups < NPC<br>RE, CN < DNA<br>RE < CL |
| Executive functioning (EXIT); n=253 | 7.16 (3.22) | 8.68 (3.77) | 7.36 (4.92) | 6.61 (3.62) | 5.20 (2.43) | 5.47 | 4, 245 | 0.30 | .009 | NPC<CL,CN |

| **Total N = 285**<br>*Variables also presented in the main article are **bolded*** | **Careless Labile Attempters [CL]**<br>n = 71<br>means (SD) | **Callous Narcissistic Attempters [CN]**<br>n = 25<br>mean (SD) | **Rigid Extraverted Attempters [RE]**<br>n = 13<br>mean (SD) | **Depressed Non-Attempter Comparisons [DNA]**<br>n = 111<br>mean (SD) | **Non-Psychiatric Comparisons [NPC]**<br>n = 65<br>mean (SD) | **Test statistic**<br>*F-statistic unless other test specified* | **Degrees of freedom** | **Effect size**<br>*Cohen's f unless otherwise specified* | **Adjusted *p*-value**<br>*Benjamini-Hochberg correction for 29 tests* | **Tukey-adjusted post hoc pairwise contrasts** |
|---|---|---|---|---|---|---|---|---|---|---|
| **Impulsivity (BIS); n=281** | 70.06 (11.43) | 70.08 (12.76) | 67.54 (12.68) | 65.65 (9.48) | 51.89 (7.64) | 34.97 | 4, 273 | 0.72 | <.001 | DNA<CL<br>NPC<other groups |
| **Anger rumination (ARS); n=284** | 38.33 (10.86) | 39.20 (11.54) | 29.62 (7.03) | 35.60 (9.14) | 24.48 (4.70) | 26.01 | 4, 276 | 0.61 | <.001 | RE,NPC< CL,CN<br>NPC<DNA |
| **Physical health** (tests adjusted for age and sex) | | | | | | | | | | |
| **Physical illness burden (CIRS-G) – median [IQR]; n=270** | 9 [5-13] | 8 [5.25-11.75] | 9 [6-14.5] | 9 [6-13] | 4.5 [2-6] | 15.41[d] | 4, 263 | 0.47 | <.001 | NPC<other groups |
| Severe physical illness (score of 3 or 4 in any organ system; CIRS-G) – count (%); n=270 | 25 (35.21%) | 7 (28.00%) | 3 (23.08%) | 28 (25.22%) | 5 (7.69%) | 16.35[a] | 4, 263 | 0.25[d] | .075 | - |
| **Social health** (tests adjusted for age and sex) | | | | | | | | | | |
| **SNI – Network diversity – median [IQR]; n=284** | 3 [2-5] | 4 [2-5] | 5 [4-5] | 4 [3-6] | 6 [5-7] | 13.74[d] | 4, 277 | 0.44 | <.001 | CL,CN,DNA<NPC |
| SNI – Number of people – median [IQR]; n=284 | 7 [3.25-11] | 8 [4-9] | 9 [7-15] | 9 [4-13.5] | 13 [9-19] | 10.73[d] | 4, 277 | 0.38 | <.001 | CL,CN,DNA<NPC |
| SNI – Embedded network – median [IQR]; n=284 | 0 [0-1] | 0 [0-1] | 1 [1-1] | 1 [0-2] | 1 [1-2] | 7.94[d] | 4, 277 | 0.34 | <.001 | CL,CN,DNA<NPC |
| **Childhood Trauma (CTQ) – median [IQR]; n=269** | 51 [37-63.5] | 48 [35.5-65] | 33.5 [26.75-49.25] | 44.5 [33.75-52.25] | 28 [25-33] | 84.22[d] | 4, 262 | 0.62 | <.001 | NPC< CL,CN,DNA;<br>RE,DNA<CL |

*Note.* Difference compared to the main analysis: the effect size highlighted in pink no longer corresponds to a strong effect size based on its Cramer's V value (see *Adjusting for socio-demographic covariates* in the Results for more details). Dark grey columns indicate attempter profiles identified by LPA; light grey columns indicate non-attempter comparison groups. Values in all grey columns represent mean (standard deviation) unless otherwise specified. Differences having a large effect size (Cohen's f ≥ 0.40 or Cramer's V ≥ 0.6) are bolded. P-values have been adjusted for 29 tests using Benjamini-Hochberg's method. The number of observations is specified under variables with missing data. Legend: IQR, interquartile range; NEO-FFI, NEO Five-Factor Inventory; BPNI-G, Brief Pathological Narcissism Inventory – Grandiosity subscale; PAI-BOR, Personality Assessment Inventory-Borderline Scale; HDRS, Hamilton Rating Scale for Depression; SSI; Beck Scale of Suicidal Ideation; SCID, Structured Clinical Interview for DSM-IV disorders; MMSE, Mini Mental State Exam; DRS, Global Cognitive Mattis Dementia Rating Scale; BIS, Barratt Impulsivity Scale; ARS, Anger Rumination Scale; CIRS-G, Cumulative Illness Rating Scale – Geriatrics; SNI, Social Network Index; CTQ, Childhood Trauma Questionnaire.

[a-c], Likelihood ratio chi-square statistic obtained from: [a]binomial logistic regression, [b]multinomial logistic regression, or [c]negative binomial logistic regression; [d], Parametric ANCOVA on the ranks; [e], Effect size computed as Cramer's V.

**Supplemental Table S10**

*Adjusted analyses covarying for age and sex (and education in the case of cognitive functioning) of the change from baseline to two-years follow-up in suicidal behavior, suicidal ideation severity, depression severity, physical illness burden, and cognitive functioning by depressed study groups*

| **Total sample at two-year visit:**<br>**N = 172** | **Careless Labile Attempters [CL]**<br>**n = 61** | **Callous Narcissistic Attempters [CN]**<br>**n = 19** | **Rigid Extraverted Attempters [RE]**<br>**n = 11** | **Depressed Non-Attempter Comparisons [DNA]**<br>**n = 81** | **Test statistic**<br>*F-statistic unless otherwise specified* | **Degrees of freedom** | **Effect size**<br>*Cohen's f unless otherwise specified* | ***p*-value** | **Post hoc pairwise contrasts**<br>*Adjusted with Tukey* |
|---|---|---|---|---|---|---|---|---|---|
| Any follow-up suicidal behavior (vs none) – count (%) [a]<br>n = 91 | 12<br>(19.67%) | 3<br>(15.79%) | 1<br>(9.09%) | - | 0.86[a] | 2, 86[b] | 0.10 | .649 | - |
| Mean change in worst suicidal ideation severity (SSI)<br>n = 140 | -3.20 (11.08) | -5.62 (14.79) | -8.83 (11.37) | 0.31<br>(3.87) | 4.03 | 3, 134 | 0.30 | .009 | no pairwise differences |
| **Mean change in depression severity (HDRS)**<br>**n = 147** | -6.42 (7.36) | -7.20<br>(8.35) | -15.40 (9.56) | -5.98<br>(6.77) | 2.71 | 3, 141 | 0.24 | .048 | RE < CL, DNA |
| **Mean change in cognitive functioning (MMSE) [#]**<br>**n = 126** | 0.33<br>(1.58) | -0.27<br>(2.05) | -1.88<br>(3.76) | 0.28<br>(0.95) | 4.96 | 3, 119 | 0.35 | .003 | RE < CL, DNA |
| Mean change in physical illness burden (CIRS-G)<br>n = 132 | 0.60<br>(2.73) | 1.46<br>(1.45) | -0.50<br>(1.64) | 1.08<br>(2.73) | 1.07 | 3, 126 | 0.16 | .365 | - |

*Note*. All tests covaried for age and sex, whereas the analysis for mean change in cognitive functioning ([#]) covaried for education as well. Dark grey columns indicate attempter profiles identified by LPA within the attempter group; the light grey column indicates the depressed non-attempter comparison group. Numbers in the grey cells represent mean (SD) unless otherwise specified. Positive scores indicate an increase from baseline and negative scores a decrease from baseline. Higher scores indicate more unfavorable outcomes for all variables except for cognitive functioning. Variables with significant pairwise group differences after Tukey's adjustment are bolded and match the results of the unadjusted analysis (Table 3). Number of observations is indicated for each variable as follow-up assessments were missing in some participants. Legend: SSI, Beck Scale of Suicidal Ideation; HDRS, Hamilton Rating Scale for Depression; CIRS-G, Cumulative Illness Rating Scale – Geriatrics; MMSE, Mini Mental State Exam.

[a], Likelihood ratio chi-square statistic obtained from binomial logistic regression; [b], Effect size computed as Cramer's V.
